# From the Balkan towards Western Europe: Range expansion of the golden jackal (*Canis aureus*)—A climatic niche modeling approach

**DOI:** 10.1002/ece3.9141

**Published:** 2022-07-24

**Authors:** Sarah Cunze, Sven Klimpel

**Affiliations:** ^1^ Institute of Ecology, Evolution and Diversity Goethe‐University Frankfurt/Main Germany; ^2^ Senckenberg Biodiversity and Climate Research Centre Senckenberg Gesellschaft für Naturforschung Frankfurt/Main Germany

**Keywords:** biomod, climate change‐induced range shifts, ensemble forecasting consensus model, parasites, predator pressure, species distribution modeling

## Abstract

In recent decades, a rapid range expansion of the golden jackal (*Canis aureus*) towards Northern and Western Europe has been observed. The golden jackal is a medium‐sized canid, with a broad and flexible diet. Almost 200 different parasite species have been reported worldwide from *C. aureus*, including many parasites that are shared with dogs and cats and parasite species of public health concern. As parasites may follow the range shifts of their host, the range expansion of the golden jackal could be accompanied by changes in the parasite fauna in the new ecosystems. In the new distribution area, the golden jackal could affect ecosystem equilibrium, e.g., through changed competition situations or predation pressure. In a niche modeling approach, we project the future climatic habitat suitability of the golden jackal in Europe in the context of whether climatic changes promote range expansion. We use an ensemble forecast based on six presence‐absence algorithms to estimate the climatic suitability of *C. aureus* for different time periods up to the year 2100 considering different IPCC scenarios on future development. As predictor variables, we used six bioclimatic variables provided by worldclim. Our results clearly indicate that areas with climatic conditions analogous to those of the current core distribution area of the golden jackal in Europe will strongly expand towards the north and the west in future decades. Thus, the observed range expansion may be favored by climate change. The occurrence of stable populations can be expected in Central Europe. With regard to biodiversity and public health concerns, the population and range dynamics of the golden jackal should be surveyed. Correlative niche models provide a useful and frequently applied tool for this purpose. The results can help to make monitoring more efficient by identifying areas with suitable habitat and thus a higher probability of occurrence.

## Graphical abstract

In recent decades, a rapid range expansion of the golden jackal (Canis aureus) towards northern and western Europe has been observed. In an ensemble forecasting niche modelling approach, we projected the potential future range expansion of the golden jackal in Europe.

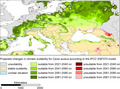

## 1 INTRODUCTION

In the face of global change, many species have been observed to alter their distributional ranges and further changes are expected over the next decades (Dormann, 2007; Parmesan, 2006). These changes are the result of a variety of different drivers and mechanisms. Changes in climatic conditions are certainly of great relevance in this context (Parmesan & Yohe, 2003). Intensification of agriculture and changes in land use and land cover causing habitat losses are other contributing factors (Agariga et al., 2021; Egli et al., 2018; Smeraldo et al., 2020). In addition, global trade and tourism have caused biogeographical boundaries to be dissolved, making it easier for species to migrate to regions that have been inaccessible before (Hobbs et al., 2006). As a result, species are expected to expand beyond their historic ranges and may occur in new relative abundances and combinations (Hobbs et al., 2006). The introduction of new species, or any change in species composition at all, leads to changes in biotic interactions in terms of parasites, predators, and competitors (Bertolino et al., 2020; Dormann, 2007; Mayr et al., 2020; Parmesan & Yohe, 2003). This leads to modified biocenoses and altered ecosystems (Hobbs et al., 2006; Jenssen et al., 2021).

The golden jackal (*Canis aureus*) is one of the species that has recently observed to alter its range (Jirků et al., 2018). For a long time, the distribution of the golden jackal remained restricted to the southeast of Eurasia (Arnold et al., 2012). Since the 19th century, strong population fluctuations occurred with drastic declines, leading to local extinctions and strong increases accompanied by a rapid range expansion. The range expansion mainly started in the second half of the 20th century from the Balkans along the Danube basin towards Central Europe (Arnold et al., 2012; Trouwborst et al., 2015) and is probably still ongoing. It remains still unclear which factors promote or drive this area’s expansion (reported in Gherman & Mihalca, 2017; Trouwborst et al., 2015).

Meanwhile, *C. aureus* is a widespread species in Eurasia with a distribution focus in the South East (i.e., the Balkans, Anatolia, and Caucasus). The current distributional range covers Arabian Peninsula, Middle East, and Caucasus eastwards into the Indian subcontinent and Southeast Asia with the highest population densities to be found in the central and eastern part of Balkan Peninsula (Penezić & Ćirović, 2015). The golden jackal has been reported in 33 European countries (Hatlauf et al., 2021) with established populations in about 20 European countries (reported in Trouwborst et al., 2015; Gherman & Mihalca, 2017).

The golden jackal is a medium‐sized opportunistic mesopredator with a broad range of food categories, including small mammals (mainly rodents), birds (and their eggs), amphibians, reptiles, invertebrates, and sporadically plants (especially fruits) (Farkas et al., 2017; Gherman & Mihalca, 2017; Lange et al., 2021; Lanszki et al., 2016; Penezić & Ćirović, 2015; Torretta et al., 2021). This flexible and generalist diet of the golden jackal is supposed to promote the currently observed area expansion (Hatlauf et al., 2021). Its opportunistic omnivorous diet makes *C. aureus* a potential food competitor with the red fox (*Vulpes vulpes*; Farkas et al., 2017; Lanszki et al., 2016). Thus, Giannatos (2004) notes that the population densities of fox and golden jackal may strongly interact with each other. Lanszki and Heltai (2010) on the other hand, state that the available biomass of small mammals in Europe is sufficient for both species to coexist without resource partitioning, especially in view of the flexible diet of both species.

Golden jackals are suspected to avoid areas where wolves (top predators) occur. The decline of the wolf population in Central and Western Europe is therefore considered as a possible further aspect that promotes the westward spread of the golden jackal (Krofel et al., 2017; Spassov & Acosta‐Pankov, 2019). On a large scale, the ranges of both species may overlap and this is the case in parts of southwestern Europe (Chapron et al., 2014; Krofel et al., 2017), where the wolf is currently subjected to range dynamics as well (Szewczyk et al., 2019).

Interacting with numerous other species (parasites, prey, and competitors) the golden jackal is part of complex food webs, predator–prey relationships, and host–parasite relationships (Gherman & Mihalca, 2017; Lange et al., 2021; Lanszki et al., 2016). The species plays a prominent role in the ecosystem as a canid mesopredator. Thus, its range expansion may lead to considerable changes in ecosystems.

This aspect is seen controversially in the literature. Hatlauf et al. (2021), state that so far no negative effects of the jackal on the ecosystem are known. However, in Bulgaria, where the jackal occurs in very high abundance, the species is supposed to cause significant economic damage due to game losses (Stoyanov, 2012).

Implicated in the epidemiological cycle of a vast variety of zoonotic pathogens, the new occurrence of the species in substantial abundances could be of health concern. In total, 194 parasite species have been reported in golden jackals (Gherman & Mihalca, 2017), including *Echinococcus* spp., hookworms, *Toxocara* spp., or *Trichinella* spp., which have a high zoonotic potential (Gherman & Mihalca, 2017) and are thus of high public health relevance. The high diversity of parasites in the golden jackal is attributed to the species' wide geographic range, extensive territorial mobility, large home ranges, and broad food spectrum (Gherman & Mihalca, 2017)—this is likely to be similar to other mesopredators such as foxes and badgers. Changes in spatial patterns may also involve the ranges and local abundances of closely interacting species. Thus, parasite species of the golden jackal might follow its range expansions. Certain parasite species may be newly introduced in this area, and the abundance and prevalences of other species may change. For example, Balog et al. (2021) suggest that this could be the case for the human pathogenic helminths *Echinococcus multilocularis* and *Echinococcus granulosus* s.l., which may also enhance their European range or local abundances. The enhanced emergence of *Dirofilaria immitis* in Hungary over the last 12 years is associated with the rising abundance of the golden jackal as an important reservoir host for the heartworm (Széll et al., 2020). As a canid species, the golden jackal is of particular parasitological interest, as many of its parasites may also infect domestic dogs (Gherman & Mihalca, 2017). The golden jackal occupies a variety of habitats, including proximity to humans (Amouei et al., 2018; Meshgi et al., 2009; Nabavi et al., 2014). The diverse parasite fauna, including parasite species shared with dogs and cats, and human pathogenic species, underline the health relevance of *C. aureus* and the importance of addressing its distribution patterns.

### 1.1 Aims and scope

Here, we estimated the potential range expansion of the golden jackal in Europe. Based on a niche modeling approach, we projected the climatic habitat suitability for *C. aureus* under current and future climatic conditions. For future conditions, we considered four shared socioeconomic pathways (SSPs) provided by the Intergovernmental Panel on Climate Change (IPCC), accounting for different scenarios of future development based on different climate policies.

## 2 MATERIAL AND METHODS

### 2.1 Distributional data

We assembled distributional information for the golden jackal in Europe from various sources and compiled these into a map (Figure S1). On the one hand, these are point data based on individual observations, on the other hand, polygon data describing the core distribution area. In particular, occurrence records (point data) were taken from the Global Biodiversity Information Facility GBIF (www.gbif.org) (https://doi.org/10.15468/dl.fzbjxe, 14,003 records) and from vertnet (www.vertnet.org, 510 records), both as of 3.12.2021, and literature (Arnold et al., 2012; Hatlauf et al., 2021; Kowalczyk et al., 2020; Männil & Ranc, 2022; Rykov et al., 2022; Sørensen & Lindsø, 2021; Trouwborst et al., 2015; Zagorodniuk, 2014). Polygon data was provided by the International Union for Conservation of Nature (IUCN, Hoffmann et al., 2018) and in literature (Arnold et al., 2012; Trouwborst et al., 2015).

We excluded point data from Africa for the following reason: Recent phylogenetic studies have shown that at least two of the African subspecies should be recognized as distinct species (Gherman & Mihalca, 2017). Koepfli et al. (2015) postulate that *C. aureus anthus*, with a distributional range in northern Africa (Hoffmann & Atickem, 2019), forms a separate monophyletic lineage to *C. aureus* and should be recognized as a separate species. The Egyptian jackal (*C. aureus lupaster*) seems to be phylogenetically more closely related to other wolf‐like canids from the gray wolf species complex than to subspecies of the golden jackal and should therefore also be considered separately (Knispel Rueness et al., 2011). In the present study, the European golden jackal (*C. aureus moreoticus*) is considered.

The available point data (Figure S1) seem to be strongly biased, over‐representing the edge of the core distribution. These observations probably refer to vagrant individuals rather than to established occurrences (Arnold et al., 2012) and, because they are considered somewhat exceptional, are more likely to be reported to, e.g., GBIF, which may explain this bias. We therefore decided to base the modeling mainly on polygon data, which seem to be more reliable and provide a better representation of the climatic niche. We use point data within the core distribution area complementarily. Point data outside the core distribution area were used to evaluate the projected recent range expansion.

Based on the polygon data describing the observed core distribution of the golden jackal we draw randomly pseudo‐presences for model training. We considered polygon data from three sources (Arnold et al., 2012; Hoffmann et al., 2018; Trouwborst et al., 2015). These polygons differ slightly from each other on a small scale but coincide on a large scale. To take this variability into account, we have drawn the pseudo‐presences separately from each polygon. Consequently, we obtained a kind of consensus data set: Areas in which the polygon data of several sources coincide are represented by more pseudo‐presences. The polygons from Arnold et al. (2012) and Trouwborst et al. (2015) were represented by 100 pseudo‐presences, the IUCN map (Hoffmann et al., 2018), which is available for the entire range of *C. aureus*, in contrast to the previous ones, with 1000 pseudo‐absences.

We restricted the study area to the northwest of the range of the golden jackal with an extent of 35° North to 60° North and 5° West to 50° East, as most of the sources of distribution data we considered relate to this area.

The pseudo‐presences derived from the polygon data were supplemented by point data from the above‐mentioned sources with the following restrictions: (a) records from 1970 onwards, (b) records within the IUCN range (Hoffmann et al., 2018), and (c) records that are not too closely located.

For the latter step, we applied the R package spThin (Aiello‐Lammens et al., 2015). All occurrence data were thinned out with the minimum distance set to 50 km in order to reduce spatial autocorrelation and to counteract the unbalanced distribution of occurrence data. This means that the minimum distance between two occurrences in the final data set is at least 50 km. This largely exceeds the home range, which is given with different sizes in the literature, e.g., 11.2 km^2^ on average with a range of 1.3–32.5 km^2^ according to Fenton et al. (2021) or about 47.1 ± 2.5 km^2^ according to Kamler et al. (2021).

The final data set for modeling contains 387 records within the study area (Figure S2). We randomly split this data into a test and training data set in a 70:30 ratio.

### 2.2 Predictor variables

We used data on climatic conditions provided by worldclim (version 2.0, www.worldclim.org; Fick & Hijmans, 2017). Nineteen so‐called bioclimatic variables are available referring to the climatic conditions (monthly temperature and precipitation) empirically recorded over a period of 30 years from 1970 to 2000. For model training, we chose a subset of six variables that are only slightly correlated with one another. We calculated the Pearson correlation coefficients for each pair of the 19 bioclim variables using the function *cor* of R's stats package (R Development Core Team, 2021) and clustered them in a dendrogram using the dissimilarity measure 1−|*r*_P_| and a threshold of 0.3 (i.e., |*r*_P_| < .7, see Figure S3). This is the most commonly applied threshold to reduce collinearity in the environmental data sets (Dormann et al., 2013). We then excluded bio08, bio09, bio18, and bio19, which combine temperature and precipitation information in one layer. They show weird spatial discontinuities that are presumably not existent in the environmental conditions and should therefore be interpreted as anomalies (Ruiz Barlett et al., 2019).

From each cluster with several intercorrelated variables (Figure S3), we chose one representative that we consider to be ecologically relevant or that appears to be easy to interpret. Specifically, we chose isothermality (bio03), temperature seasonality (bio04), maximum temperature of the warmest month (bio05), minimum temperature of the coldest month (bio06), annual precipitation (bio12), and precipitation seasonality (bio15). These variables are only little correlated (|*r*_P_| < .7, Table S1) among each other.

Data on climatic conditions were downloaded at a spatial resolution of 2.5 arc minutes and cropped to the extent of 35° North to 60° North and 5° West to 50° East. For this area reliable distribution data is available.

### 2.3 Ecological niche modeling

We projected the habitat suitability for *C. aureus* under current and future climatic conditions based on an ecological niche modeling approach. We performed ensemble forecasting (EF), which applies several statistical algorithms and combines them into a consensus model (Araújo & New, 2007). This consensus model is considered a more robust estimator and thus superior to individual models (Marmion et al., 2009).

Sillero (2011) points out that problems can arise when combining the results of different approaches, e.g., approaches based on different input data. In general, one distinguishes between presence‐absence models (e.g., regression), presence‐background models (e.g., the maximum entropy approach) and presence‐only models (e.g., climate envelope approaches). If reliable information on species absence is available, presence‐absence models are preferable to presence‐only models, and presence‐background approaches. Thus, we consider the following six presence‐absence approaches from the algorithms implemented in the R package biomod2 (Thuiller, Georges, Engler, & Breiner, 2021; Thuiller, Georges, Gueguen, et al., 2021): ANN—artificial neuronal networks; GAM—generalized additive models; GBM—generalized boosted models; GLM—generalized linear models; FDA—flexible discriminant analysis; and RF—Random Forest.

Ten thousand pseudo‐absences were chosen at random, but the area close to the observed occurrences of the golden jackal was excluded (distance <80 km), as closely located sites tend to show similar environmental conditions and thus the same niche (disk strategy implemented in the biomod2 package).

The models were run using the following single algorithm parameters: For the ANN, we used five cross‐validations to find the best size and decay parameters, and set the initial random weights on [−0.1, 0.1] with 200 iterations at maximum. For GAM we used a binomial distribution and logit link function. GBM were run with a maximum of 2500 trees to ensure fitting, a minimum number of observations in trees' terminal nodes of 10, a learning rate of 0.01, and an interaction depth of 7. To generate the GLM, we applied a stepwise feature selection with quadratic terms based on the Akaike Information Criterion (AIC). The flexible discriminant analysis (FDA) was run with the method *mars*. RF was applied with 500 trees and a node size of 5.

The consensus map represents the weighted average of the single results of the six algorithms considered. Weighting is done with the respective area under the receiver‐operating characteristic curve—AUC (Fielding & Bell, 1997) value. Only those algorithms are included with an AUC exceeding a certain threshold (here 0.85).

For model evaluation, we used the AUC value as a threshold independent performance measure (Fielding & Bell, 1997), and Kappa (Cohen, 1960) and the true skill statistics (TSS; Allouche et al., 2006). Kappa is the proportion of specific agreement and can be interpreted as followed: Kappa < 0.4: poor; 0.4 < Kappa < 0.75: good; and Kappa > 0.75: excellent (Fielding & Bell, 1997).

In order to project the future climatic suitability for *C. aureus* we used data on future climate conditions according to the fifth IPCC report (IPCC, 2014) accounting for four shared socioeconomic pathways (SSPs), 1.26, 2.45, 3.70, and 5.85, processed based on the CNRM‐ESM2‐1 Global Circulation model (Seferian, 2018). Considering different SSPs, we accounted for different scenarios of future development based on different climate policies (SSP1: sustainability, SSP2: middle paths, SSP3: regional rivalry, SSP5: fossil‐fueled development).

In order to evaluate if, to what extent, and where extrapolations can occur when projecting future habitat suitability (Elith et al., 2010), we used the multivariate environmental similarity surface (MESS) method implemented in Maxent (Figure S4). The MESS analysis helps to identify areas with future climatic conditions outside the range covered by the climatic conditions used for model training (near‐current conditions of 1970–2000). Modeling results for projected habitat suitability under future conditions outside the training range (extrapolations) should be treated with strong caution.

To transform the continuous modeling results into binary ones in order to be able to display the projected changes in four‐time steps in one map (Figure 3, Figures S9–S12), we applied the threshold that minimizes the difference between sensitivity and specificity to transform (Liu et al., 2013).

The relative contribution of the six predictor variables was standardized for the six individual algorithms applied and presented in a bar plot (Figure S14).

### 2.4 Software

Spatial analysis was conducted in ESRI ArcGIS version 10.8.1 (ESRI, 2018) and in R version 4.1.2 (R Development Core Team, 2021). For the MESS analysis, we used MAXENT version 3.4.1 (Phillips et al., n.d.). Modeling was executed in the R environment (R Development Core Team, 2021). All maps were created with ESRI ArcGIS (ESRI, 2018).

## 3 RESULTS

**FIGURE 1:**
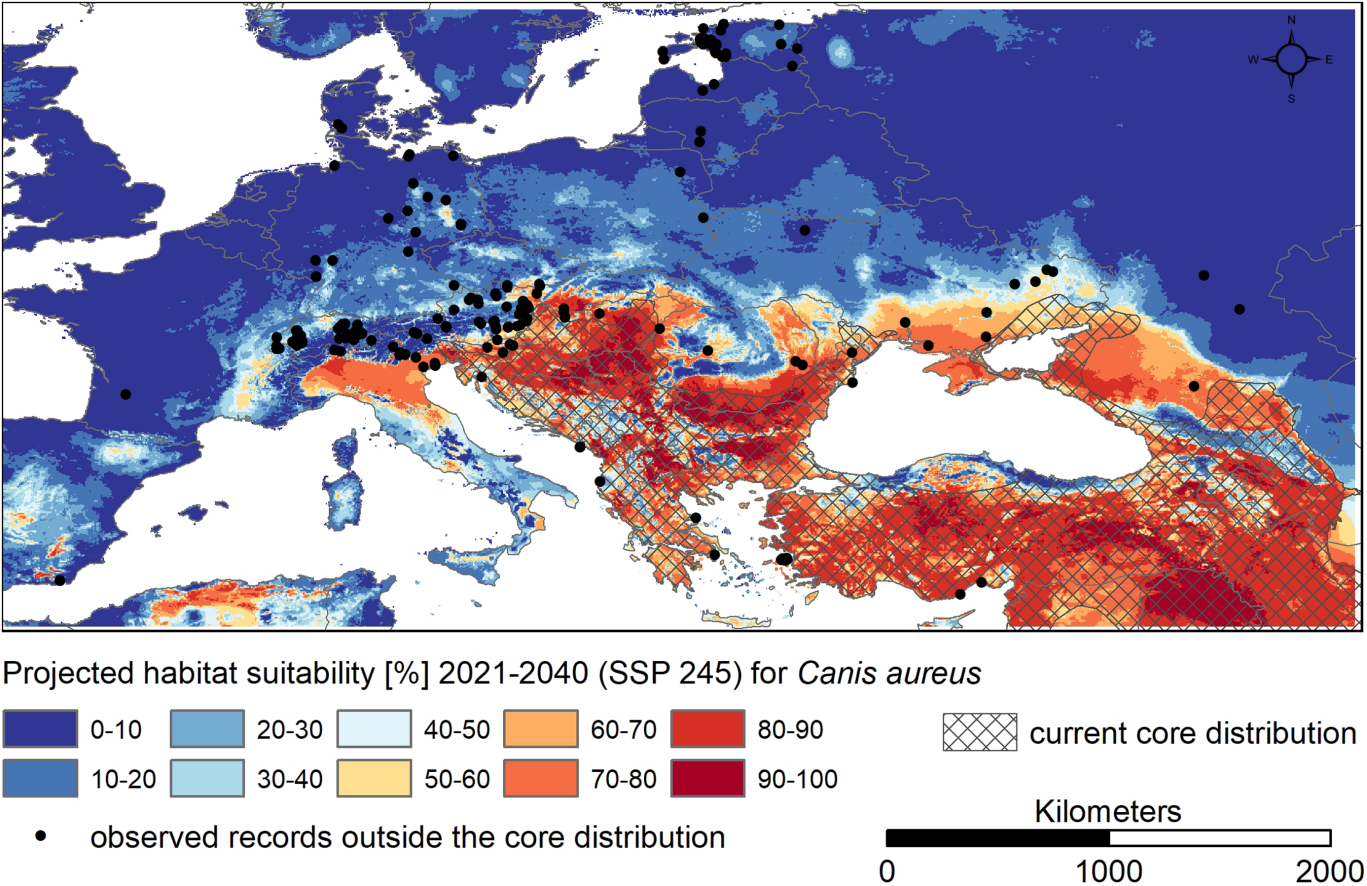
Modeled climatic suitability (ensemble model) for the golden jackal (*Canis aureus*) under near‐current climatic conditions (1970–2000). The map was created with ESRI ArcMap version 10.8.1. Coordinate system: WGS 1984.

The estimated habitat suitability by the EF consensus model under near‐current climatic conditions (1970–2000) (Figure 1) reflects the observed distribution of the golden jackal (Figure S1) very well with high modeling performance parameters (AUC = 0.977, Kappa = 0.656 and TSS = 0.858, Table S3).

**FIGURE 2:**
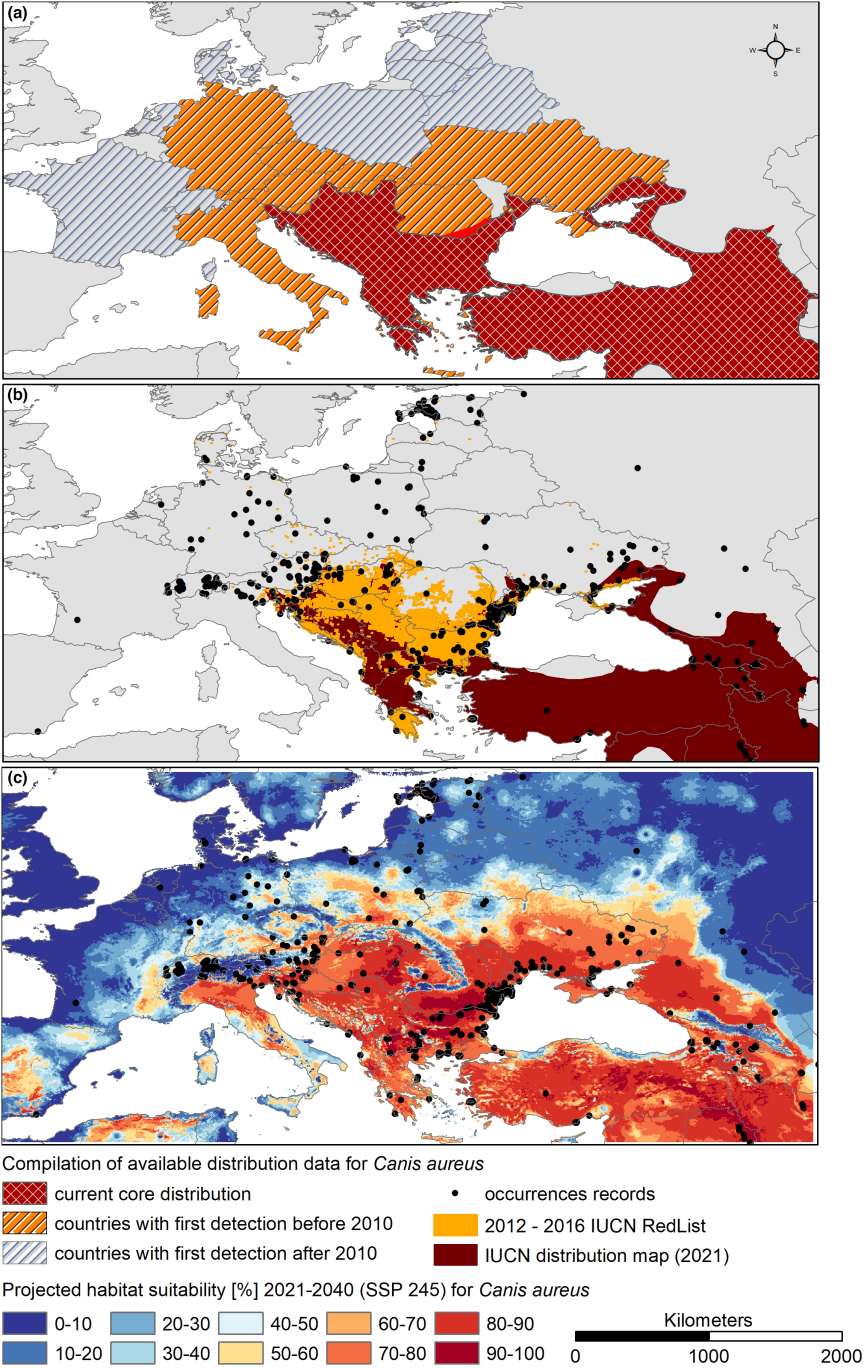
Compilation of information on the distribution of the golden jackal in Europe compared with near‐future climatic habitat suitability. (a) Species‐reported occurrence on a national scale, countries with reported first detection of *Canis aureus* before and after 2010 (Hatlauf et al., 2021), (b) core distribution (Hoffmann et al., 2018; Ranc et al., 2022), (c) modeled climatic suitability (ensemble model) for the golden jackal (*Canis aureus*) under projected near‐future climatic conditions (2021–2040, SSP 245) together with available occurrences records (Arnold et al., 2012; GBIF, 2021; Jirků et al., 2018; Kowalczyk et al., 2020; Männil & Ranc, 2022; Rykov et al., 2022; Trouwborst et al., 2015; Zagorodniuk, 2014). The map was created with ESRI ArcMap version 10.8.1. Coordinate system: WGS 1984.

Some of the records of golden jackal outside the core population, which were not included in the model training, are covered by the future projections for the period 2021–2040; others are not (Figure 2c). Most of these records date between the two periods covered by the modeling (i.e., roughly 2000–2020).

**FIGURE 3:**
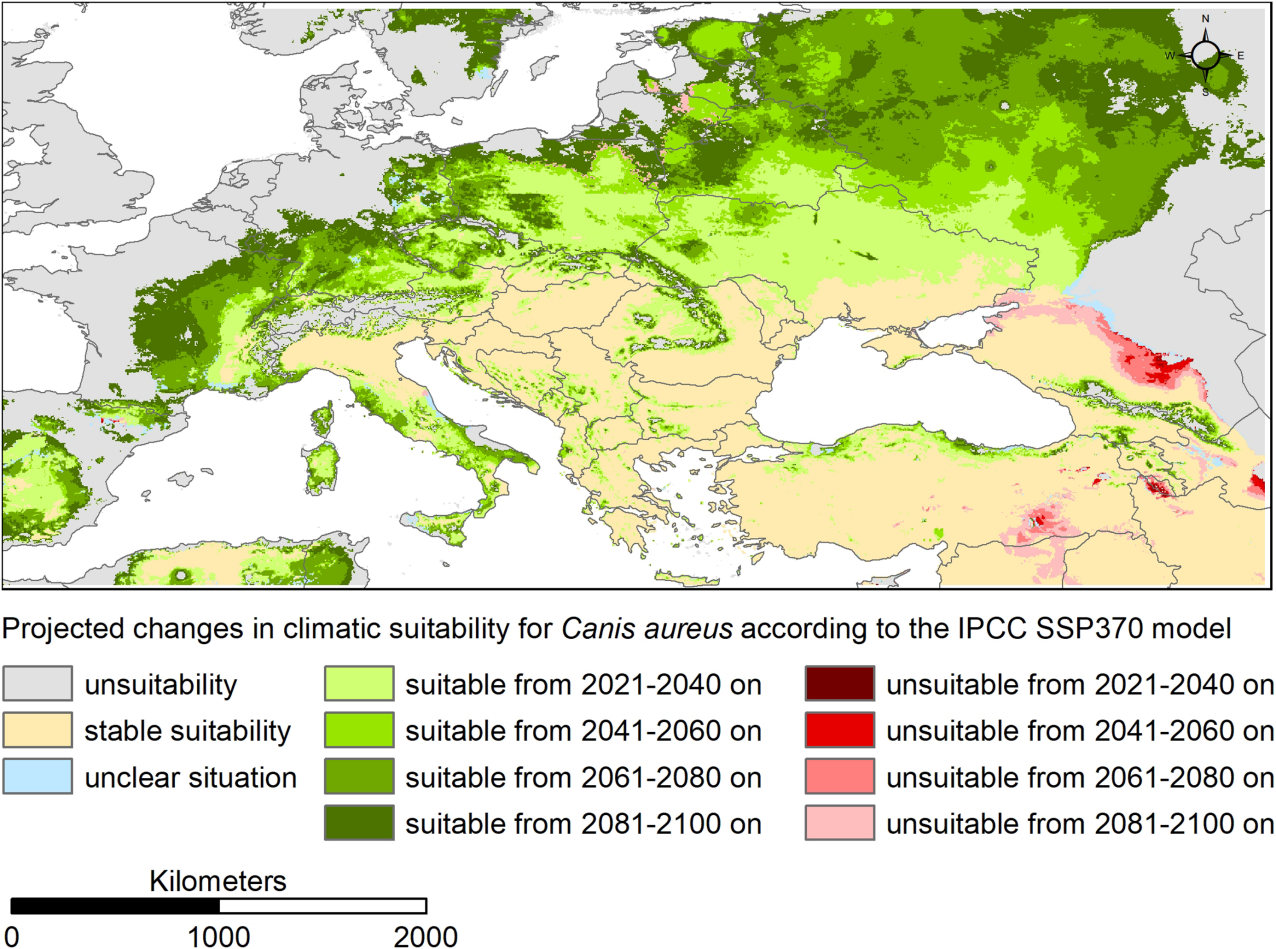
Projected changes (ensemble model) in areas of modeled climatic suitability for the golden jackal (*Canis aureus*) in four‐time steps to 2100 according to the IPCC scenario SSP 245 in relation to near‐current conditions (1970–2000). We applied the threshold (th = 0.48) that minimizes the difference between sensitivity and specificity to transform the continuous modeling results into binary ones. The map was created with ESRI ArcMap version 10.8.1. Coordinate system: WGS 1984. Further modeling results are shown in the supplement (Figures S9–S12).

**FIGURE 4:**
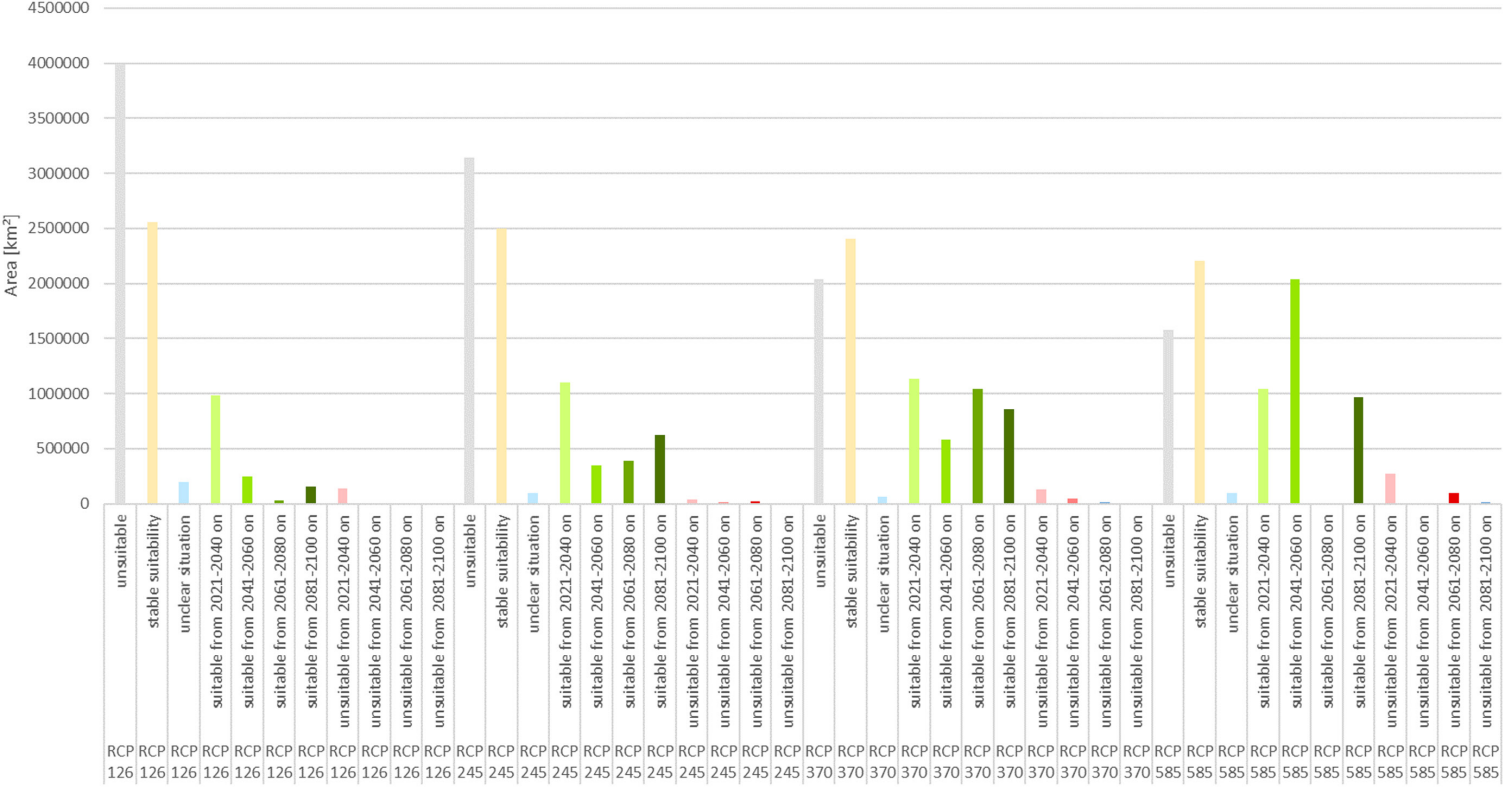
Projected areas (km^2^) modeled as climatically suitable or unsuitable for the golden jackal (*Canis aureus*)—Considering four‐time steps to 2100 in relation to near‐current conditions (1970–2000) and comparing the IPCC scenario (SSP 126, SSP 245, SSP 370, SSP 585). See Figures S9–S12 for the corresponding maps.

The future projections of climatic suitability for the golden jackal in Europe agree on showing a steady expansion towards the north and the west over the period 2021 to 2100 across all four IPCC scenarios considered (Figures 3 and 4 and Figures S5–S12). Future projections that assume greater changes in climatic conditions (i.e., SSP 585) are characterized by a greater increase in the area with modeled climatic suitability for the golden jackal (Figure 4).

Temperature variables (especially summer temperature and temperature seasonality) tend to show a higher contribution to the results of the single models than precipitation variables (Figure S14).

## 4 DISCUSSION

Using a correlative niche modeling approach, we modeled climatic habitat suitability for the golden jackal in Europe under current and projected future conditions. We conducted a state‐of‐the‐art EF approach, which combines several single models into a consensus model providing a more robust estimator (Araújo & New, 2007). Here, we consider climatic habitat suitability based on empirically collected interpolated temperature and precipitation data between 1970 and 2000. We related the core distribution of the golden jackal to these data. We only considered distribution data in the core distribution area to ensure that these are occurrences that can be attributed to established, reproductive populations at this time. Data from most recent publications that may indicate newly established occurrences outside the core distribution area (e.g., Jirků et al., 2018; Kowalczyk et al., 2020; Männil & Ranc, 2022) were not accounted for in the model training, but we displayed these observations together with near‐future projections (for the period of 2021–2040) of climatic habitat suitability for the golden jackal.

Due to specific data situation, we were able to incorporate presence‐absence algorithms, which, in the case of reliable data, include more information in the modeling. The current core distribution is well reflected in the modeled habitat suitability patterns. This is a necessary assumption, which is required to use the model for valid future projections.

In some areas, modeled habitat suitability exceeds observed distribution (e.g., northern Italy, southern Ukraine). Here, other nonclimatic factors (e.g., land use, barriers to dispersal) may act as limiting factors, especially that golden jackals expand in the areas with no suitable climate (Estonia, Finland, Norway, Russia; Jirků et al., 2018). However, this does not prevent the model from being considered overall sound, also in view of the high model performance measures (Table S3). Future projections show an expansion of the area with projected climatic suitability for the golden jackal in Europe towards the north and west (Smeraldo et al., 2021). This indicates that the currently observed range expansion of the golden jackal from the Balkans towards Western Europe is favored by climate change. At least, it can be stated that the climatic conditions in these potential new areas will become similar to those that are currently characteristic in the core distribution area of the golden jackal. However, it is difficult to say to what extent the observed and assumed further expansion of the range is mainly driven by climate change and to what extent other nonclimatic factors play a role (Spassov & Acosta‐Pankov, 2019). Land use and land cover may also play an important role, and the distributional patterns of interacting species (e.g., the gray wolf, *Canis lupus*, e.g., Giannatos, 2004; Krofel et al., 2017; Trouwborst et al., 2015) and human pressure.

Distribution patterns of species are the result of a variety of factors with climatic conditions being considered one of the most important drivers on a continental scale (Pacifici et al., 2020; Pearson, 2007).

Besides, interspecific competition is considered a key factor shaping the distribution patterns of species (Pacifici et al., 2020). Competing species with similar ecological requirements that are sympatrically distributed, may coexist or mutually exclude each other, depending on the availability of resources (Torretta et al., 2021). The presence of the gray wolf (*Canis lupus*) is considered to be an important limiting factor for the distribution of the golden jackal, as wolf occurrences can prevent the establishment of the golden jackal (Krofel et al., 2017). As the wolf is considered a natural intra‐guild predator of the golden jackal, range expansion and establishment in new areas of the latter species is suspected to be more likely in areas, where the wolf is absent or at least uncommon (Trouwborst et al., 2015). However, in the last decades, the wolf population has increased again (Chapron et al., 2014) and golden jackals have been increasingly reported in countries with strong or growing wolf populations (Poland, Finland, Estonia). In order to avoid direct contact with the wolf, the golden jackal may turn to other habitat types: wolf colonization of forest areas and golden jackal may switch to more open habitat types; this may contribute to the spatial segregation of both species at a regional scale. Thus, at the considered continental scale, the presence of the wolf level is less important.

The advantage of correlative methods for species distribution modeling is that biotic conditions are also taken into account. The models are based on observed distribution data representing the occupied niche (Pearson, 2007; Sillero, 2011; Sobéron & Peterson, 2005). The occupied niche represents the area in the niche space where both, abiotic and biotic conditions fit. Thus, correlative niche models thus incorporate biotic factors (at least indirectly). However, the results should be interpreted only in relation to the considered predictor variables. Thus, despite projected climatic suitability, a region may be excluded from the successful establishment of *C. aureus* if, for example, there is a high abundance of wolves (Giannatos, 2004; Krofel et al., 2017; Trouwborst et al., 2015), or if it is physically not reachable due to dispersal barriers.

In addition to the assumption that the ongoing climate change is contributing to the observed range changes of the golden jackal in Europe (Arnold et al., 2012; Giannatos, 2004), it is also suspected that land‐use change is of high relevance (Šálek et al., 2014; Zagorodniuk, 2014). In recent decades, much agricultural land has been abandoned in Central and Eastern Europe (Alcantara et al., 2013), creating a suitable habitat for golden jackals, which has favored their spread into Ukraine, Belarus, and further north.

We have not included land cover in our analysis (due to the lack of future scenarios). The fact that the modeled potential distribution area, e.g., in northern Italy, extends beyond the observed core distribution could possibly be justified by the fact that land cover is not included. However, it can be assumed that the golden jackal is able to cope with a wide range of conditions.

Another important factor determining the distribution patterns of species is the accessibility of areas (mobility [M] in the nomenclature of Sobéron & Peterson, 2005). In terms of the expected and already observed range expansion of the golden jackal towards Western Europe, this factor is of less importance, as there are no major physical barriers to dispersal such as oceans or mountain ranges present. Nevertheless, migration routes such as river valleys are of great relevance (Jongman et al., 2011).

We have restricted the study area to those areas where we have information on the presence or absence of the species. The fact that we consider only a part of the species' range may lead us to obtain a partial niche model. Consequently, our approach may underestimate the climatic suitability of *C. aureus*.

Records outside the core range were displayed in Figure 2b,c to evaluate near‐future range expansion. In these areas, reproduction of the golden jackal has rarely been reported so far (Kowalczyk et al., 2020; Männil & Ranc, 2022). Some of these observed point records are located in areas with modeled habitat suitability under projected near‐future climatic conditions (2021–2040, Figure 2c). It can be assumed that permanent populations may be established here. The other observations, in areas modeled as climatically unsuitable under near‐future conditions (2021–2040, Figure 2c), can be justified as animals on migration may be able to survive for some time under suboptimal climatic conditions, even if, for example, reproduction is rare. Permanent establishment at the edge of the climatic niche seems rather unlikely.

Successful range expansion and the colonization of new areas involve four processes (Estrada et al., 2016; Kowalczyk et al., 2020): (1) Emigration: individuals leave their previous territory. (2) Movement: individuals disperse over long distances (golden jackals have been observed via telemetry to be able to disperse long distances and even through human‐dominated landscapes Lanszki et al., 2018). (3) Establishment: successful reproduction takes place (for 2015 and 2017, Kowalczyk et al. (2020) reported the northernmost reproduction in Poland so far). (4) Proliferation: a certain reproduction rate is required to maintain a permanently stable population. Under optimal conditions, reproduction rates are assumed to be generally higher than under suboptimal conditions, e.g., at the edge of the niche. However, if other conditions are good or the golden jackal partially adapts to the new conditions, this is also possible. In any case, vagrant animals can indicate a spread into areas outside the core populations and are a prerequisite for this.

## 5 CONCLUSION

The new occurrence of species in an area may bring considerable changes in ecosystems and may even pose a threat to native biodiversity. The established occurrence of the golden jackal with high abundance in new areas may have an impact on the ecosystems, e.g., through altered predation pressure, including on rare and endangered species, or competitive pressure on other mesopredators (Hobbs et al., 2006; Trouwborst et al., 2015). In addition, economic losses due to killed domestic animals and game are conceivable (Stoyanov, 2012). Crossbreeding with golden jackals and wolves or domestic dogs is thought to be possible (Trouwborst et al., 2015). Parasites associated with the golden jackal could follow the migration of the host species. Due to the close relationship between golden jackals and dogs, this could also be of relevance to veterinary medicine and human medicine (Gherman & Mihalca, 2017). Implicated in the epidemiological cycle of a vast variety of zoonotic pathogens, the new occurrence of the species in substantial abundances could thus also be of health concern. Therefore, understanding the current distribution patterns, their underlying factors, and future range changes is crucial. Our results clearly indicate that climate change is likely to favor the further spread of the species the golden jackal in Europe. The present study provides a valuable contribution in that monitoring and survey programmes can be conducted more efficiently by identifying areas with higher habitat suitability and thus also a higher probability of occurrence of the golden jackal.

## AUTHOR CONTRIBUTIONS

**Sarah Cunze:** Conceptualization (lead); formal analysis (lead); methodology (lead); project administration (equal). **Sven Klimpel:** Conceptualization (supporting); funding acquisition (lead); project administration (equal).

## CONFLICT OF INTEREST

The authors declare that there is no conflict of interest.

## Supporting information


Figure S1
Figure S2
Figure S3
Table S1
Figure S4
Figure S5
Figure S6
Figure S7
Figure S8
Figure S9
Figure S10
Figure S11
Figure S12
Figure S13
Table S2
Figure S14

Table S3

## Data Availability

Occurrence data used for modeling are provided in Table S3.

## References

[ece39141-bib-0001] Agariga, F. , Abugre, S. , & Appiah, M. (2021). Spatio‐temporal changes in land use and forest cover in the Asutifi North District of Ahafo region of Ghana, (1986–2020). Environmental Challenges, 5, 100209.

[ece39141-bib-0002] Aiello‐Lammens, M. E. , Boria, R. A. , Radosavljevic, A. , Vilela, B. , & Anderson, R. P. (2015). spThin: An R package for spatial thinning of species occurrence records for use in ecological niche models. Ecography, 38(5), 541–545.

[ece39141-bib-0003] Alcantara, C. , Kuemmerle, T. , Baumann, M. , Bragina, E. V. , Griffiths, P. , Hostert, P. , Knorn, J. , Müller, D. , Prishchepov, A. V. , Schierhorn, F. , Sieber, A. , & Radeloff, V. C. (2013). Mapping the extent of abandoned farmland in central and Eastern Europe using MODIS time series satellite data. Environmental Research Letters, 8(3), 35035.

[ece39141-bib-0004] Allouche, O. , Tsoar, A. , & Kadmon, R. (2006). Assessing the accuracy of species distribution models: Prevalence, kappa and the true skill statistic (TSS). Journal of Applied Ecology, 43(6), 1223–1232.

[ece39141-bib-0005] Amouei, A. , Jahandar, H. , Daryani, A. , Sharif, M. , Sarvi, S. , Mizani, A. , Hosseini, S. A. , Sarafrazi, M. , Siyadatpanah, A. , Gohardieh, S. , Bastani, R. , & Gholami, S. (2018). Carnivores as important reservoirs of intestinal helminthic infections in Mazandaran Province, Northern Iran. Iranian Journal of Parasitology, 13(2), 251–257. 30069209 PMC6068364

[ece39141-bib-0006] Araújo, M. B. , & New, M. (2007). Ensemble forecasting of species distributions. Trends in Ecology & Evolution, 22(1), 42–47. 17011070 10.1016/j.tree.2006.09.010

[ece39141-bib-0007] Arnold, J. , Humer, A. , Heltai, M. , Murariu, D. , Spassov, N. , & Hackländer, K. (2012). Current status and distribution of golden jackals *Canis aureus* in Europe. Mammal Review, 42(1), 1–11.

[ece39141-bib-0008] Balog, T. , Nagy, G. , Halász, T. , Csányi, E. , Zomborszky, Z. , & Csivincsik, Á. (2021). The occurrence of *Echinococcus* spp. in golden jackal (*Canis aureus*) in southwestern Hungary: Should we need to rethink its expansion? Parasitology International, 80, 102214. 33137507 10.1016/j.parint.2020.102214

[ece39141-bib-0009] Bertolino, S. , Sciandra, C. , Bosso, L. , Russo, D. , Lurz, P. W. , & Di Febbraro, M. (2020). Spatially explicit models as tools for implementing effective management strategies for invasive alien mammals. Mammal Review, 50(2), 187–199.

[ece39141-bib-0010] Chapron, G. , Kaczensky, P. , Linnell, J. D. C. , von Arx, M. , Huber, D. , Andrén, H. , López‐Bao, J. V. , Adamec, M. , Álvares, F. , Anders, O. , Balčiauskas, L. , Balys, V. , Bedő, P. , Bego, F. , Blanco, J. C. , Breitenmoser, U. , Brøseth, H. , Bufka, L. , Bunikyte, R. , … Boitani, L. (2014). Recovery of large carnivores in Europe's modern human‐dominated landscapes. Science (New York, N.Y.), 346(6216), 1517–1519. 10.1126/science.1257553 25525247

[ece39141-bib-0011] Cohen, J. (1960). A coefficient of agreement of nominal scales. Educational and Psychological Measurement, 20, 37–46.

[ece39141-bib-0012] Dormann, C. F. (2007). Promising the future? Global change projections of species distributions. Basic and Applied Ecology, 8(5), 387–397.

[ece39141-bib-0013] Dormann, C. F. , Elith, J. , Bacher, S. , Buchmann, C. , Carl, G. , Carré, G. , Marquéz, J. R. G. , Gruber, B. , Lafourcade, B. , Leitão, P. J. , Münkemüller, T. , McClean, C. , Osborne, P. E. , Reineking, B. , Schröder, B. , Skidmore, A. K. , Zurell, D. , & Lautenbach, S. (2013). Collinearity: A review of methods to deal with it and a simulation study evaluating their performance. Ecography, 36(1), 27–46.

[ece39141-bib-0014] Egli, L. , Meyer, C. , Scherber, C. , Kreft, H. , & Tscharntke, T. (2018). Winners and losers of national and global efforts to reconcile agricultural intensification and biodiversity conservation. Global Change Biology, 24(5), 2212–2228. 29389056 10.1111/gcb.14076

[ece39141-bib-0015] Elith, J. , Kearney, M. , & Phillips, S. (2010). The art of modelling range‐shifting species. Methods in Ecology and Evolution, 1(4), 330–342.

[ece39141-bib-0016] ESRI . (2018). ArcGIS Desktop: Release 10.8.1. Environmental Systems Research Institute.

[ece39141-bib-0017] Estrada, A. , Morales‐Castilla, I. , Caplat, P. , & Early, R. (2016). Usefulness of species traits in predicting range shifts. Trends in Ecology & Evolution, 31(3), 190–203. 26776962 10.1016/j.tree.2015.12.014

[ece39141-bib-0018] Farkas, A. , Jánoska, F. , Fodor, J.‐T. , & Náhlik, A. (2017). The high level of nutritional niche overlap between red fox (*Vulpes vulpes*) and sympatric golden jackal (*Canis aureus*) affects the body weight of juvenile foxes. European Journal of Wildlife Research, 63(3), 1–4. 10.1007/s10344-017-1101-x

[ece39141-bib-0019] Fenton, S. , Moorcroft, P. R. , Ćirović, D. , Lanszki, J. , Heltai, M. , Cagnacci, F. , Breck, S. , Bogdanović, N. , Pantelić, I. , Ács, K. , & Ranc, N. (2021). Movement, space‐use and resource preferences of European golden jackals in human‐dominated landscapes: Insights from a telemetry study. Mammalian Biology, 101(5), 619–630.

[ece39141-bib-0020] Fick, S. E. , & Hijmans, R. J. (2017). WorldClim 2: New 1‐km spatial resolution climate surfaces for global land areas. International Journal of Climatology, 37(12), 4302–4315.

[ece39141-bib-0021] Fielding, A. H. , & Bell, J. F. (1997). A review of methods for the assessment of prediction errors in conservation presence/absence models. Environmental Conservation, 24(1), 38–49.

[ece39141-bib-0022] GBIF . (2021). *Occurrence download: For* Canis aureus. The Global Biodiversity Information Facility.

[ece39141-bib-0023] Gherman, C. M. , & Mihalca, A. D. (2017). A synoptic overview of golden jackal parasites reveals high diversity of species. Parasites & Vectors, 10(1), 419. 28915831 10.1186/s13071-017-2329-8 PMC5603039

[ece39141-bib-0024] Giannatos, G. (2004). *Conservation action plan for the golden jackal* Canis aureus *L. in Greece* . *WWF Greece* .

[ece39141-bib-0025] Hatlauf, J. , Bayer, K. , Trouwborst, A. , & Hackländer, K. (2021). New rules or old concepts? The golden jackal (*Canis aureus*) and its legal status in Central Europe. European Journal of Wildlife Research, 67. 10.1007/s10344-020-01454-2

[ece39141-bib-0026] Hobbs, R. J. , Arico, S. , Aronson, J. , Baron, J. S. , Bridgewater, P. , Cramer, V. A. , Epstein, P. R. , Ewel, J. J. , Klink, C. A. , Lugo, A. E. , Norton, D. , Ojima, D. , Richardson, D. M. , Sanderson, E. W. , Valladares, F. , Vilà, M. , Zamora, R. , & Zobel, M. (2006). Novel ecosystems: Theoretical and management aspects of the new ecological world order. Global Ecology and Biogeography, 15(1), 1–7.

[ece39141-bib-0027] Hoffmann, M. , Arnold, J. , Duckworth, J. W. , Jhala, Y. , Kamler, J. F. , & Krofel, M. (2018). Canis aureus (errata version published in 2020). The IUCN Red List of Threatened Species 2018: e.T118264161A163507876. 10.2305/IUCN.UK.2018-2.RLTS.T118264161A163507876.en

[ece39141-bib-0028] Hoffmann, M. , & Atickem, A. (2019). Canis lupaster. The IUCN Red List of Threatened Species 2019: e.T118264888A118265889. 10.2305/IUCN.UK.2019-1.RLTS.T118264888A118265889.en

[ece39141-bib-0029] IPCC . (2014). Climate change 2014: Synthesis report . Contribution of Working Groups I, II and III to the fifth assessment report of the Intergovernmental Panel on Climate Change.

[ece39141-bib-0030] Jenssen, M. , Nickel, S. , Schütze, G. , & Schröder, W. (2021). Reference states of forest ecosystem types and feasibility of biocenotic indication of ecological soil condition as part of ecosystem integrity and services assessment. Environmental Sciences Europe, 33. 10.1186/s12302-021-00458-2

[ece39141-bib-0031] Jirků, M. , Dostál, D. , Robovský, J. , & Šálek, M. (2018). Reproduction of the golden jackal (*Canis aureus*) outside current resident breeding populations in Europe: Evidence from the Czech Republic. Mammalia, 82(6), 592–595.

[ece39141-bib-0032] Jongman, R. H. G. , Bouwma, I. M. , Griffioen, A. , Jones‐Walters, L. , & van Doorn, A. M. (2011). The pan European ecological network: PEEN. Landscape Ecology, 26(3), 311–326.

[ece39141-bib-0033] Kamler, J. F. , Minge, C. , Rostro‐García, S. , Gharajehdaghipour, T. , Crouthers, R. , In, V. , Pay, C. , Pin, C. , Sovanna, P. , & Macdonald, D. W. (2021). Home range, habitat selection, density, and diet of golden jackals in the Eastern Plains landscape, Cambodia. Journal of Mammalogy, 102(2), 636–650. 34621142 10.1093/jmammal/gyab014 PMC8491366

[ece39141-bib-0034] Knispel Rueness, E. , Asmyhr, M. G. , Sillero‐Zubiri, C. , Macdonald, D. W. , Bekele, A. , Atickem, A. , & Stenseth, N. C. (2011). The cryptic African wolf: *Canis aureus lupaster* is not a golden jackal and is not endemic to Egypt. PLoS One, 6(1), e16385. 21298107 10.1371/journal.pone.0016385 PMC3027653

[ece39141-bib-0035] Koepfli, K.‐P. , Pollinger, J. , Godinho, R. , Robinson, J. , Lea, A. , Hendricks, S. , Schweizer, R. M. , Thalmann, O. , Silva, P. , Fan, Z. , Yurchenko, A. A. , Dobrynin, P. , Makunin, A. , Cahill, J. A. , Shapiro, B. , Álvares, F. , Brito, J. C. , Geffen, E. , Leonard, J. A. , … Wayne, R. K. (2015). Genome‐wide evidence reveals that African and Eurasian Golden jackals are distinct species. Current Biology, 25(16), 2158–2165. 26234211 10.1016/j.cub.2015.06.060

[ece39141-bib-0036] Kowalczyk, R. , Wudarczyk, M. , Wójcik, J. M. , & Okarma, H. (2020). Northernmost record of reproduction of the expanding golden jackal population. Mammalian Biology, 100(1), 107–111.

[ece39141-bib-0037] Krofel, M. , Giannatos, G. , Ćirovič, D. , Stoyanov, S. , & Newsome, T. M. (2017). Golden jackal expansion in Europe: A case of mesopredator release triggered by continent‐wide wolf persecution? Hystrix, The Italian Journal of Mammalogy, 28(1), 9–15.

[ece39141-bib-0038] Lange, P. N. A. M. J. G. , Lelieveld, G. , & de Knegt, H. J. (2021). Diet composition of the golden jackal *Canis aureus* in south‐East Europe – A review. Mammal Review, 51(2), 207–213.

[ece39141-bib-0039] Lanszki, J. , & Heltai, M. (2010). Food preferences of golden jackals and sympatric red foxes in European temperate climate agricultural area (Hungary). Mammalia, 74(3), 267–273.

[ece39141-bib-0040] Lanszki, J. , Kurys, A. , Szabó, L. , Nagyapáti, N. , Porter, L. B. , & Heltai, M. (2016). Diet composition of the golden jackal and the sympatric red fox in an agricultural area (Hungary). Folia Zoologica, 65(4), 310–322.

[ece39141-bib-0041] Lanszki, J. , Schally, G. , Heltai, M. , & Ranc, N. (2018). Golden jackal expansion in Europe: First telemetry evidence of a natal dispersal. Mammalian Biology, 88, 81–84.

[ece39141-bib-0042] Liu, C. , White, M. , & Newell, G. (2013). Selecting thresholds for the prediction of species occurrence with presence‐only data. Journal of Biogeography, 40(4), 778–789.

[ece39141-bib-0043] Männil, P. , & Ranc, N. (2022). Golden jackal (*Canis aureus*) in Estonia: Development of a thriving population in the boreal ecoregion. Mammal Research, 67(2), 245–250.

[ece39141-bib-0044] Marmion, M. , Hjort, J. , Thuiller, W. , & Luoto, M. (2009). Statistical consensus methods for improving predictive geomorphology maps. Computers & Geosciences, 35(3), 615–625.

[ece39141-bib-0045] Mayr, A. V. , Peters, M. K. , Eardley, C. D. , Renner, M. E. , Röder, J. , & Steffan‐Dewenter, I. (2020). Climate and food resources shape species richness and trophic interactions of cavity‐nesting Hymenoptera. Journal of Biogeography, 47(4), 854–865.

[ece39141-bib-0046] Meshgi, B. , Eslami, A. , Bahonar, A. R. , Kharrazian‐Moghadam, M. , & Gerami‐Sadeghian, A. (2009). Prevalence of parasitic infections in the red fox (*Vulpes vulpes*) and golden jackal (*Canis aureus*) in Iran. Iranian Journal of Veterinary Research, 10(4), 387–391.

[ece39141-bib-0047] Nabavi, R. , Manouchehri Naeini, K. , Zebardast, N. , & Hashemi, H. (2014). Epidemiological study of gastrointestinal helminthes of canids in Chaharmahal and Bakhtiari Province of Iran. Iranian Journal of Parasitology, 9(2), 276–281. 25848396 PMC4386050

[ece39141-bib-0048] Pacifici, M. , Rondinini, C. , Rhodes, J. R. , Burbidge, A. A. , Cristiano, A. , Watson, J. E. M. , Woinarski, J. C. Z. , & Di Marco, M. (2020). Global correlates of range contractions and expansions in terrestrial mammals. Nature Communications, 11(1), 2840. 10.1038/s41467-020-16684-w PMC7275054 32504033

[ece39141-bib-0049] Parmesan, C. (2006). Ecological and evolutionary responses to recent climate change. Annual Review of Ecology, Evolution, and Systematics, 37(1), 637–669.

[ece39141-bib-0050] Parmesan, C. , & Yohe, G. (2003). A globally coherent fingerprint of climate change impacts across natural systems. Nature, 421(6918), 37–42. 12511946 10.1038/nature01286

[ece39141-bib-0051] Pearson, R. G. (2007). Species' distribution modeling for conservation educators and practitioners. Synthesis. American Museum of Natural History, 50, 54–89.

[ece39141-bib-0052] Penezić, A. , & Ćirović, D. (2015). Seasonal variation in diet of the golden jackal (*Canis aureus*) in Serbia. Mammal Research, 60(4), 309–317.

[ece39141-bib-0053] Phillips, S. J. , Dudík, M. , & Schapire, R. E. (n.d.). [Internet] Maxent software for modeling species niches and distributions (Version 3.4.1). Retrieved from http://biodiversityinformatics.amnh.org/open_source/maxent/

[ece39141-bib-0054] R Development Core Team . (2021). R: A language and environment for statistical computing: Version 4.1.2. The R Foundation for Statistical Computing.

[ece39141-bib-0055] Ranc, N. , Acosta‐Pankov, I. , Balys, V. , Bučko, J. , Cirovic, D. , Fabijanić, N. , Filacorda, S. , Giannatos, G. , Guimaraes, N. , Hatlauf, J. , Heltai, M. , Ionescu, O. , Ivanov, G. , Jansman, H. , Kowalczyk, R. , Krofel, M. , Kutal, M. , Lanszki, J. , Lapini, L. , … Zimmermann, F. (2022). *Distribution of large carnivores in Europe 2012–2016: Distribution map for Golden jackal (*Canis aureus*)* . Zenodo.

[ece39141-bib-0056] Ruiz Barlett, T. , Martin, G. M. , Laguna, M. F. , Abramson, G. , & Monjeau, A. (2019). Climatic constraints and the distribution of Patagonian mice. Journal of Mammalogy, 100(6), 1979–1991.

[ece39141-bib-0057] Rykov, A. M. , Kuznetsova, A. S. , & Tirronen, K. F. (2022). The first record of the golden jackal (Canis aureus Linnaeus, 1758) in the Russian subarctic. Polar Biology, 45(5), 965–970.

[ece39141-bib-0058] Šálek, M. , Červinka, J. , Banea, O. C. , Krofel, M. , Ćirović, D. , Selanec, I. , Penezić, A. , Grill, S. , & Riegert, J. (2014). Population densities and habitat use of the golden jackal (*Canis aureus*) in farmlands across the Balkan Peninsula. European Journal of Wildlife Research, 60(2), 193–200.

[ece39141-bib-0059] Seferian, R. (2018). CNRM‐CERFACS CNRM‐ESM2‐1 model output prepared for CMIP6 CMIP. Earth System Grid Federation.

[ece39141-bib-0060] Sillero, N. (2011). What does ecological modelling model? A proposed classification of ecological niche models based on their underlying methods. Ecological Modelling, 222(8), 1343–1346.

[ece39141-bib-0061] Smeraldo, S. , Bosso, L. , Fraissinet, M. , Bordignon, L. , Brunelli, M. , Ancillotto, L. , & Russo, D. (2020). Modelling risks posed by wind turbines and power lines to soaring birds: The black stork (*Ciconia nigra*) in Italy as a case study. Biodiversity and Conservation, 29(6), 1959–1976.

[ece39141-bib-0062] Smeraldo, S. , Bosso, L. , Salinas‐Ramos, V. B. , Ancillotto, L. , Sánchez‐Cordero, V. , Gazaryan, S. , & Russo, D. (2021). Generalists yet different: Distributional responses to climate change may vary in opportunistic bat species sharing similar ecological traits. Mammal Review, 51(4), 571–584.

[ece39141-bib-0063] Sobéron, J. , & Peterson, A. T. (2005). Interpretation of models of fundamental ecological niches and species' distributional areas. Biodiversity Informatics, 2, 1–10.

[ece39141-bib-0064] Sørensen, O. , & Lindsø, L. (2021). The golden jackal *Canis aureus* detected in Norway – Management challenges with naturally dispersed species new to the country. Fauna, 74, 74–87.

[ece39141-bib-0065] Spassov, N. , & Acosta‐Pankov, I. (2019). Dispersal history of the golden jackal (*Canis aureus moreoticus* Geoffroy, 1835) in Europe and possible causes of its recent population explosion. Biodiversity Data Journal, 7, e34825. 31139003 10.3897/BDJ.7.e34825 PMC6522460

[ece39141-bib-0066] Stoyanov, S. (2012). *Golden jackal (*Canis aureus*) in Bulgaria, current status, distribution, demography and diet: Proceedings. [International Symposium on Hunting] Modern Aspects of Sustainable Management of Game Population, Zemun‐Belgrade, Serbia, 22–24 June, 2012, Đorđević, N. (ed. in chief).‐ Zemun (Serbia): Faculty of Agriculture, 2012*. International Symposium on Hunting Modern Aspects of Sustainable Management of Game Population, Zemun ‐ Belgrade (Serbia), 22–24 Jun 2012. Conference.

[ece39141-bib-0067] Széll, Z. , Bacsadi, Á. , Szeredi, L. , Nemes, C. , Fézer, B. , Bakcsa, E. , Kalla, H. , Tolnai, Z. , & Sréter, T. (2020). Rapid spread and emergence of heartworm resulting from climate and climate‐driven ecological changes in Hungary. Veterinary Parasitology, 280, 109067. 32145530 10.1016/j.vetpar.2020.109067

[ece39141-bib-0068] Szewczyk, M. , Nowak, S. , Niedźwiecka, N. , Hulva, P. , Špinkytė‐Bačkaitienė, R. , Demjanovičová, K. , Bolfíková, B. Č. , Antal, V. , Fenchuk, V. , Figura, M. , Tomczak, P. , Stachyra, P. , Stępniak, K. M. , Zwijacz‐Kozica, T. , & Mysłajek, R. W. (2019). Dynamic range expansion leads to establishment of a new, genetically distinct wolf population in Central Europe. Scientific Reports, 9(1), 19003. 31831858 10.1038/s41598-019-55273-w PMC6908625

[ece39141-bib-0069] Thuiller, W. , Georges, D. , Engler, R. , & Breiner, F. (2021). biomod2: Ensemble platform for species distribution modeling . R package version, 3.5.1 (3), r539.

[ece39141-bib-0070] Thuiller, W. , Georges, D. , Gueguen, M. , Engler, R. , & Breiner, F. (2021). Package ‘biomod2’: Version 3.5.1 .

[ece39141-bib-0071] Torretta, E. , Riboldi, L. , Costa, E. , Delfoco, C. , Frignani, E. , & Meriggi, A. (2021). Niche partitioning between sympatric wild canids: The case of the golden jackal (*Canis aureus*) and the red fox (*Vulpes vulpes*) in North‐Eastern Italy. BMC Ecology and Evolution, 21(1), 129. 34157980 10.1186/s12862-021-01860-3 PMC8218446

[ece39141-bib-0072] Trouwborst, A. , Krofel, M. , & Linnell, J. D. C. (2015). Legal implications of range expansions in a terrestrial carnivore: The case of the golden jackal (*Canis aureus*) in Europe. Biodiversity and Conservation, 24(10), 2593–2610.

[ece39141-bib-0073] Zagorodniuk, I. (2014). Golden jackal (*Canis aureus*) in Ukraine: Modern expansion and status of species. Proceedings of the National Museum of Natural History, 12, 100–105.

