# Supplementary material for: From the Balkan towards Western Europe: Range expansion of the golden jackal (Canis aureus)—A climatic niche modeling approach
Source: Ecol Evol. 2022 Jul 24;12(7):e9141. doi: 10.1002/ece3.9141 (PMC9309039; doi:10.1002/ece3.9141)
Supplement: Supplementary file 1 — Figure S1 Figure S2 Figure S3 Table S1 Figure S4 Figure S5 Figure S6 Figure S7 Figure S8 Figure S9 Figure S10 Figure S11 Figure S12 Figure S13 Table S2 Figure S14 [file ECE3-12-e9141-s001.docx]

# Distributional data


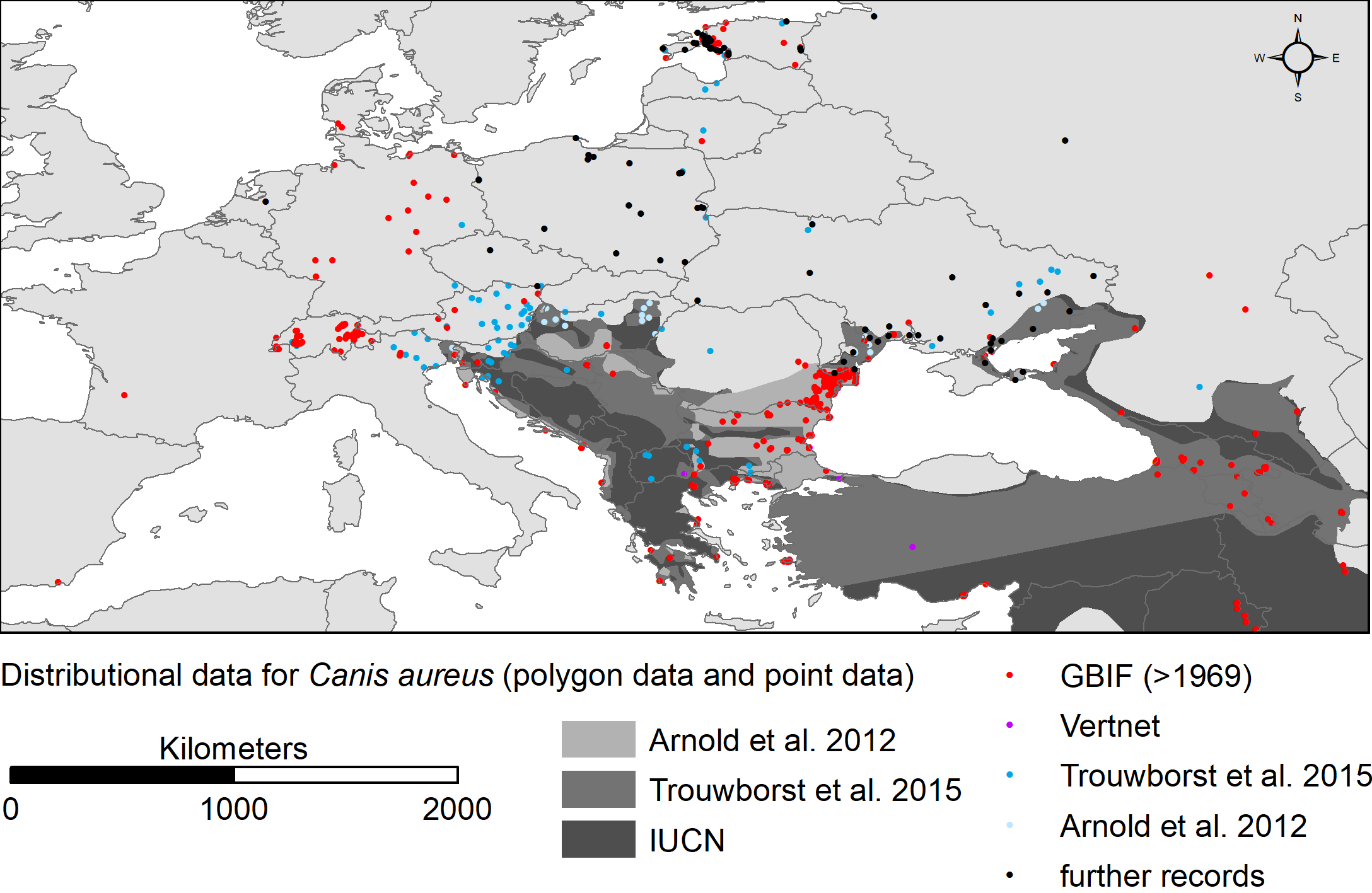


Figure S1: Distributional data available for the golden jackal (*Canis aureus*): Polygon data from Arnold et al., 2012; Trouwborst et al., 2015 and the IUCN (Hoffman et al., 2020) (Arnold *et al.* 2012; Trouwborst, Krofel & Linnell 2015; Hoffmann *et al.* 2020), Point records from GBIF (www.gbif.org (GBIF 2021)), vertnet (www.vertnet.org), Arnold et al., 2012, Trouwborst et al., 2015 and further records (Zagorodniuk 2014; Kowalczyk et al. 2015; Jirků et al. 2018; Sørensen & Lindsø 2021b; Männil & Ranc 2022; Rykov, Kuznetsova & Tirronen 2022). The map was created with ESRI ArcMap version 10.8.1. Coordinate system: WGS 1984


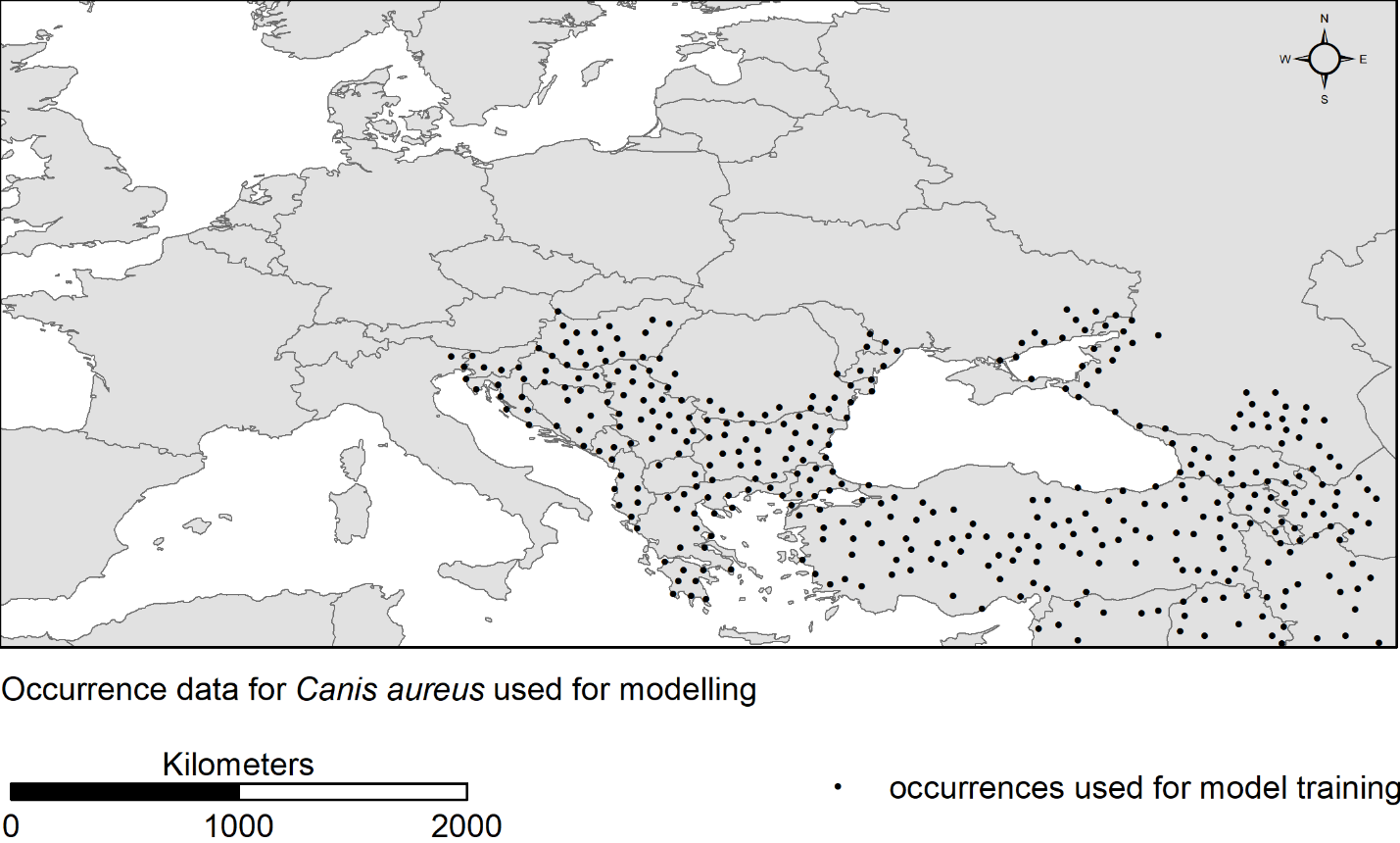
 Figure S2: Occurrence data for the Golden jackal (*Canis aureus*) used for model training. Data were derived from the information shown in Figure S1. See material and method section of the main text for details. The map was created with ESRI ArcMap version 10.8.1. Coordinate system: WGS 1984

# Variable selection


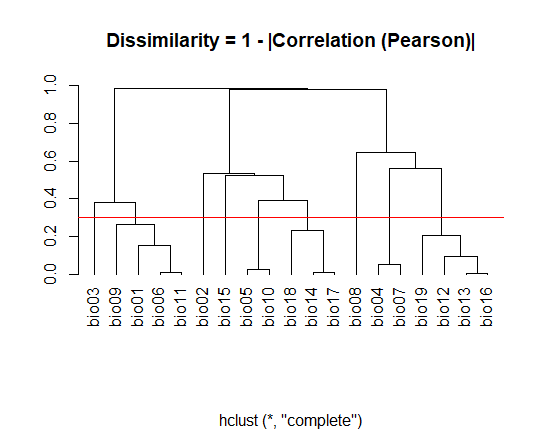


Figure S3: Dendrogram showing the collinearity of the bioclim variables (www.worldclim.org (Fick & Hijmans 2017)). We consider a threshold of 0.7 for the magnitude of the Pearson correlation coefficient, which is the most commonly used threshold (Dormann *et al.* 2013). From each cluster of intercorrelated variables, we chose one representative that we considered ecologically relevant or easy to interpret. Our chosen seven variables are: bio03 (Isothermality), bio04 (Temperature Seasonality), bio05 (Max Temperature of Warmest Month), bio06 (Min Temperature of Coldest Month), bio12 (Annual Precipitation), bio15 (Precipitation Seasonality). The remaining variables are: bio01 (Annual Mean Temperature), bio02 (Mean Diurnal Range), bio07 (Temperature Annual Range), bio08 (Mean Temperature of Wettest Quarter), bio09 (Mean Temperature of Driest Quarter), bio10 (Mean Temperature of Warmest Quarter), bio11 (Mean Temperature of Coldest Quarter), bio13 (Precipitation of Wettest Month), bio14 (Precipitation of Driest Month), bio16 (Precipitation of Wettest Quarter), bio17 (Precipitation of Driest Quarter), bio18 (Precipitation of Warmest Quarter), bio19 (Precipitation of Coldest Quarter). See material and method section of the main text for further details.

Table S1: Pearson correlation coefficients of the bioclimatic variables used for model training in the study area

|  | bio03 | bio04 | bio05 | bio06 | bio12 | bio15 |
| --- | --- | --- | --- | --- | --- | --- |
| bio03 | 1 | -0.73007637 | 0.26176427 | 0.74993583 | 0.24078305 | 0.19685407 |
| bio04 | -0.73007637 | 1 | 0.26973648 | -0.78866713 | -0.52131539 | 0.06516473 |
| bio05 | 0.26176427 | 0.26973648 | 1 | 0.326175 | -0.52193394 | 0.51143062 |
| bio06 | 0.74993583 | -0.78866713 | 0.326175 | 1 | 0.17712871 | 0.19165845 |
| bio12 | 0.24078305 | -0.52131539 | -0.52193394 | 0.17712871 | 1 | -0.18379397 |
| bio15 | 0.19685407 | 0.06516473 | 0.51143062 | 0.19165845 | -0.18379397 | 1 |

# Multidimensional environmental similarity surface (MESS) analysis and additional modelling results


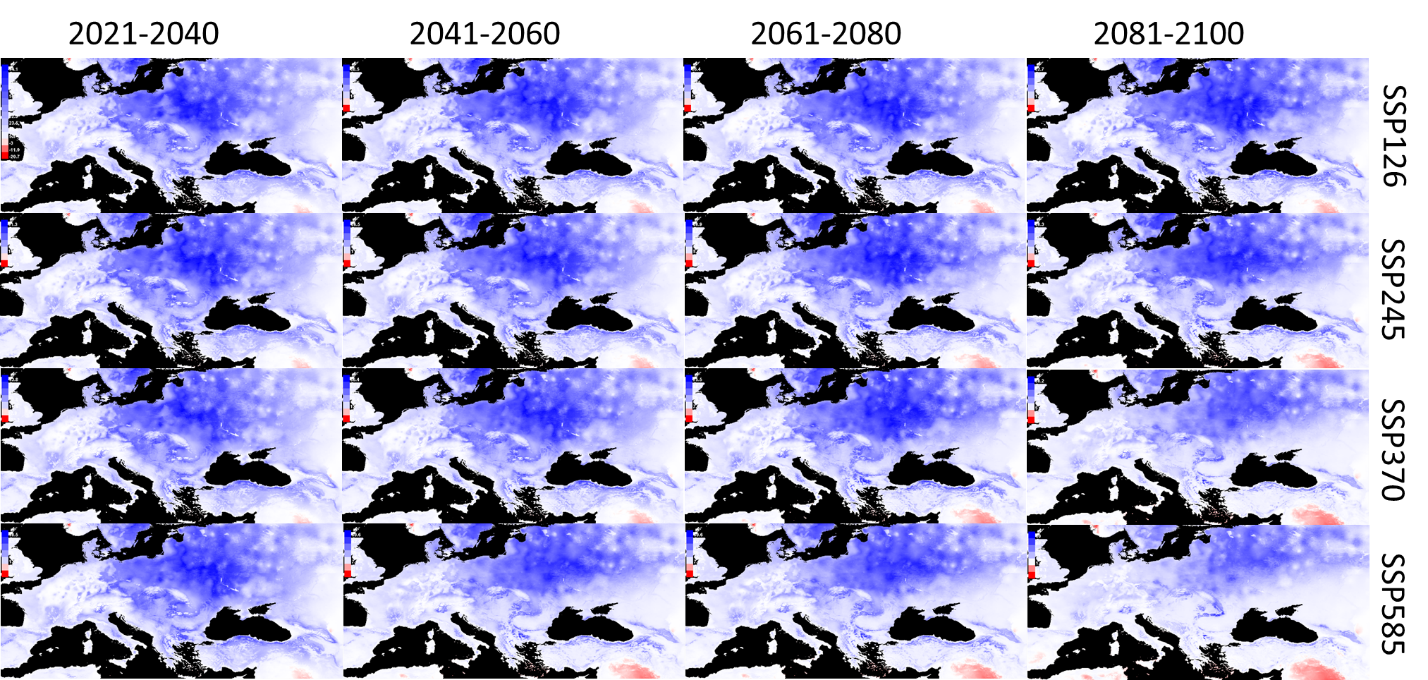


Figure S4: Results of the Maxent MESS analysis. Multidimensional environmental similarity surface (MESS) analysis indicates areas where novel climate conditions exist in the projection layers. Areas in red have one or more bioclimatic variables outside the range covered by the near current climatic conditions (1970 – 2000) used for model training. Modelling results in these areas should be treated with strong caution. Projection: WGS 1984.


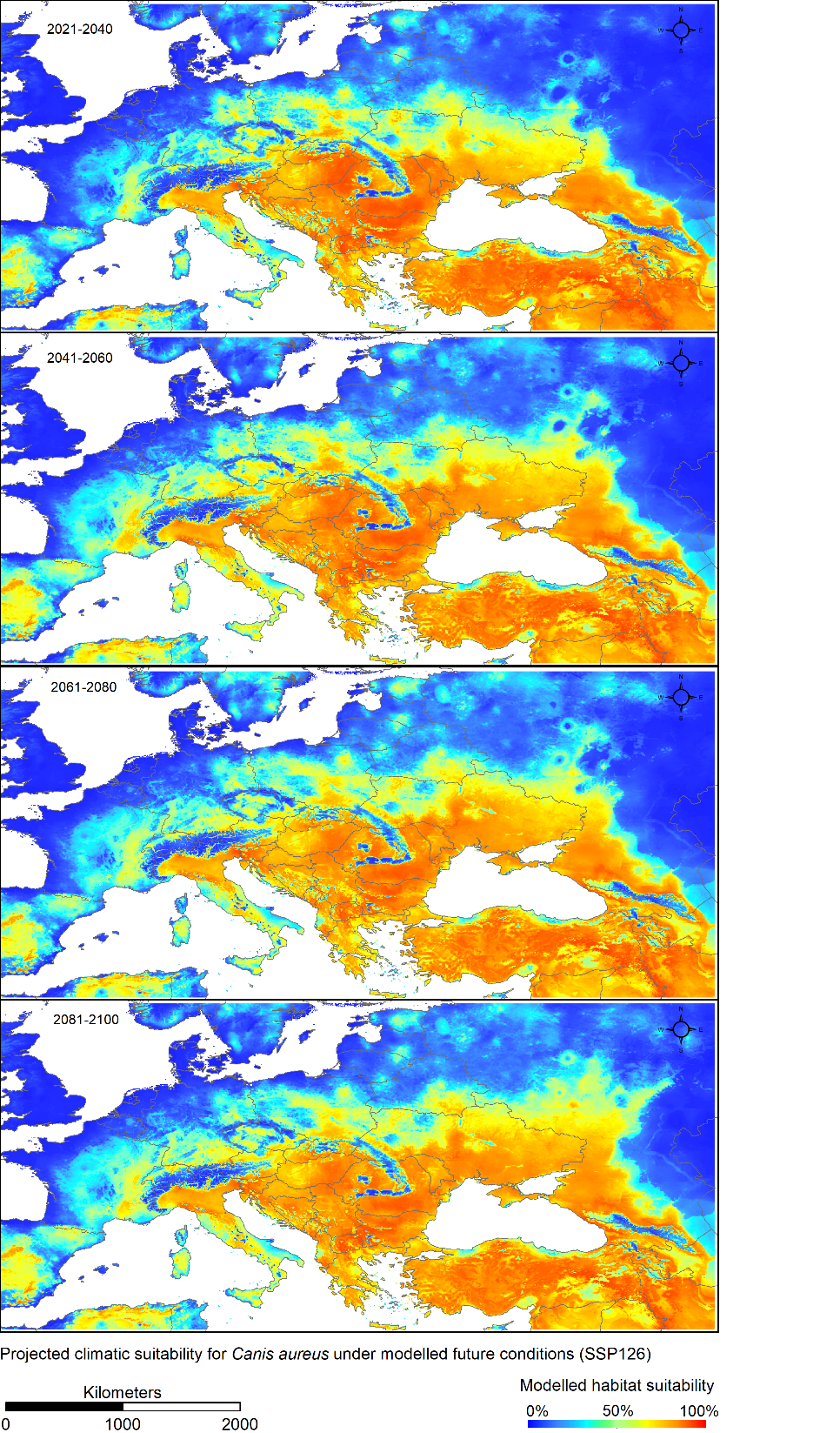


Figure S5: Modelled climatic suitability (ensemble model) for the Golden jackal (*Canis aureus*) under projected future conditions by the IPCC Scenario SSP126*.* a) for the time period 2021 – 2040, b) for the time period 2041 – 2060, c) for the time period 2061 – 2080, d) for the time period 2081 – 2100. The map was created with ESRI ArcMap version 10.8.1. Coordinate system: WGS 1984


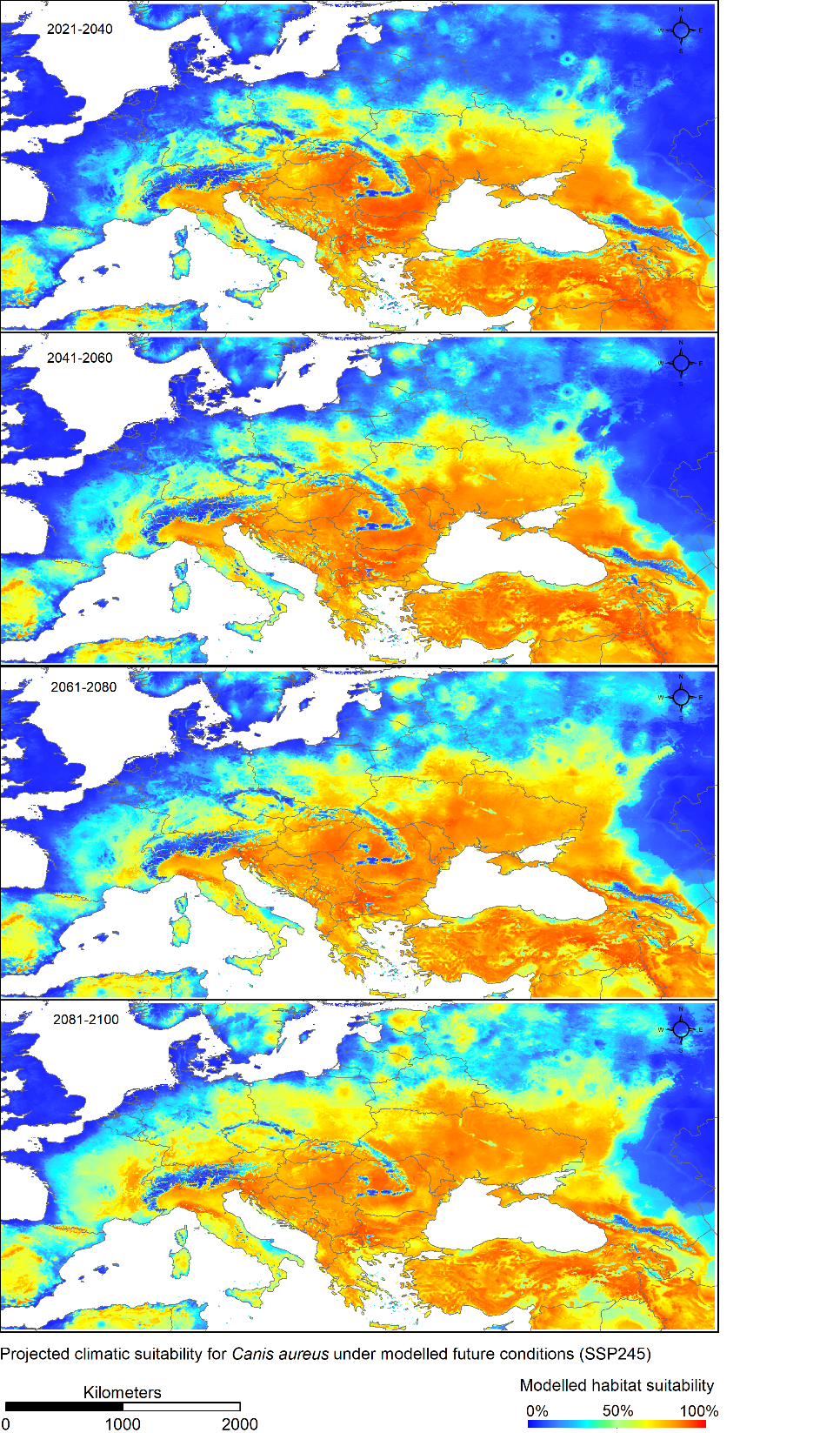


Figure S6: Modelled climatic suitability (ensemble model) for the golden jackal (*Canis aureus*) under projected future conditions by the IPCC Scenario SSP245*.* a) for the time period 2021 – 2040, b) for the time period 2041 – 2060, c) for the time period 2061 – 2080, d) for the time period 2081 – 2100. The map was created with ESRI ArcMap version 10.8.1. Coordinate system: WGS 1984


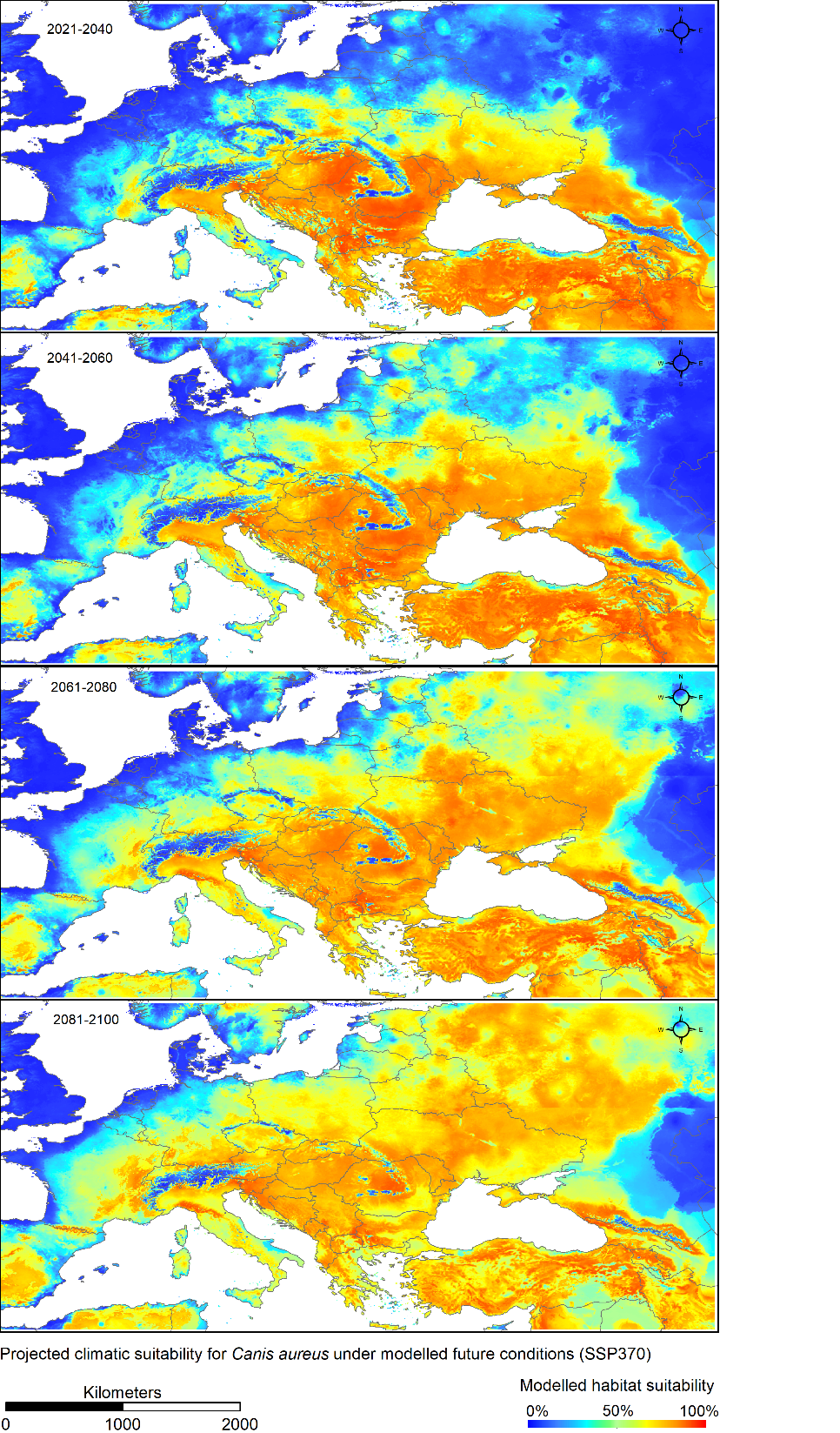


Figure S7: Modelled climatic suitability (ensemble model) for the Golden jackal (*Canis aureus*) under projected future conditions by the IPCC Scenario SSP370*.* a) for the time period 2021 – 2040, b) for the time period 2041 – 2060, c) for the time period 2061 – 2080, d) for the time period 2081 – 2100. The map was created with ESRI ArcMap version 10.8.1. Coordinate system: WGS 1984


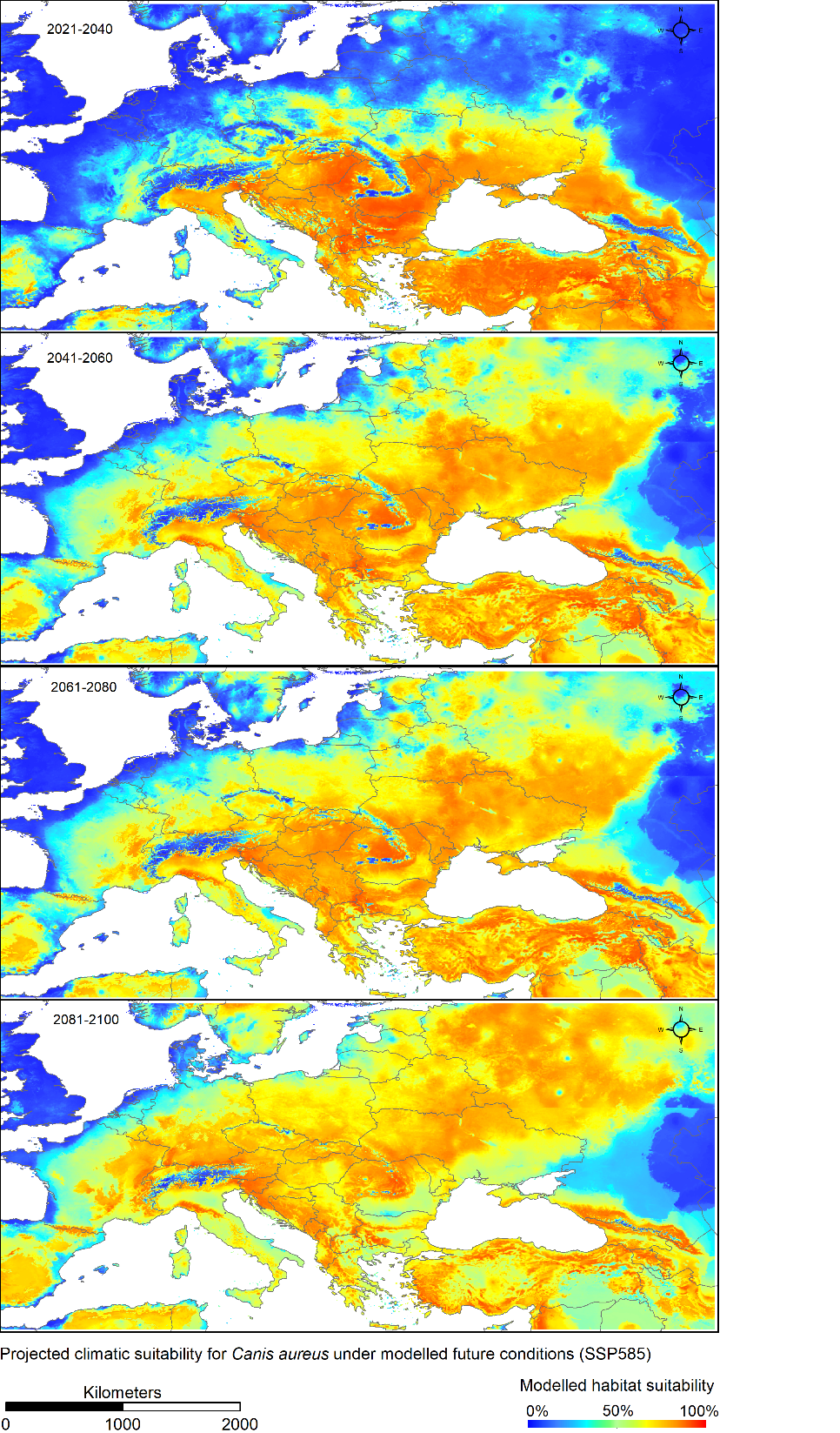


Figure S8: Modelled climatic suitability (ensemble model) for the golden jackal (*Canis aureus*) under projected future conditions by the IPCC Scenario SSP585*.* a) for the time period 2021 – 2040, b) for the time period 2041 – 2060, c) for the time period 2061 – 2080, d) for the time period 2081 – 2100. The map was created with ESRI ArcMap version 10.8.1. Coordinate system: WGS 1984


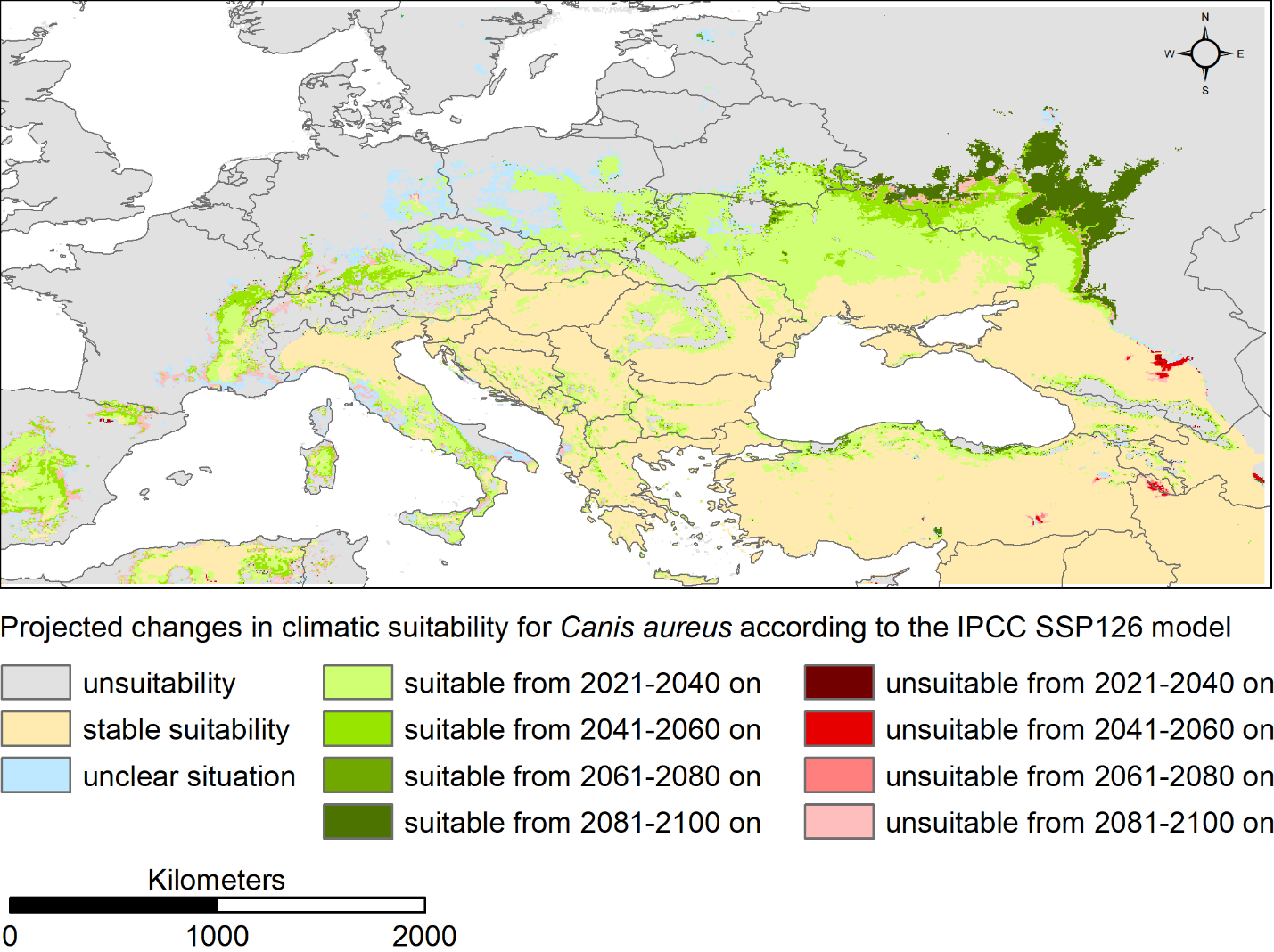


Figure S9: Projected changes (ensemble model) in areas of modelled climatic suitability for the golden jackal (*Canis aureus*) in four time steps to 2100 according to the IPCC scenario SSP 126 in relation to near current conditions (1970 - 2000). We applied the threshold (th=0.48) that minimizes the difference between sensitivity and specificity to transform the continuous modelling results into binary ones. The map was created with ESRI ArcMap version 10.8.1. Coordinate system: WGS 1984


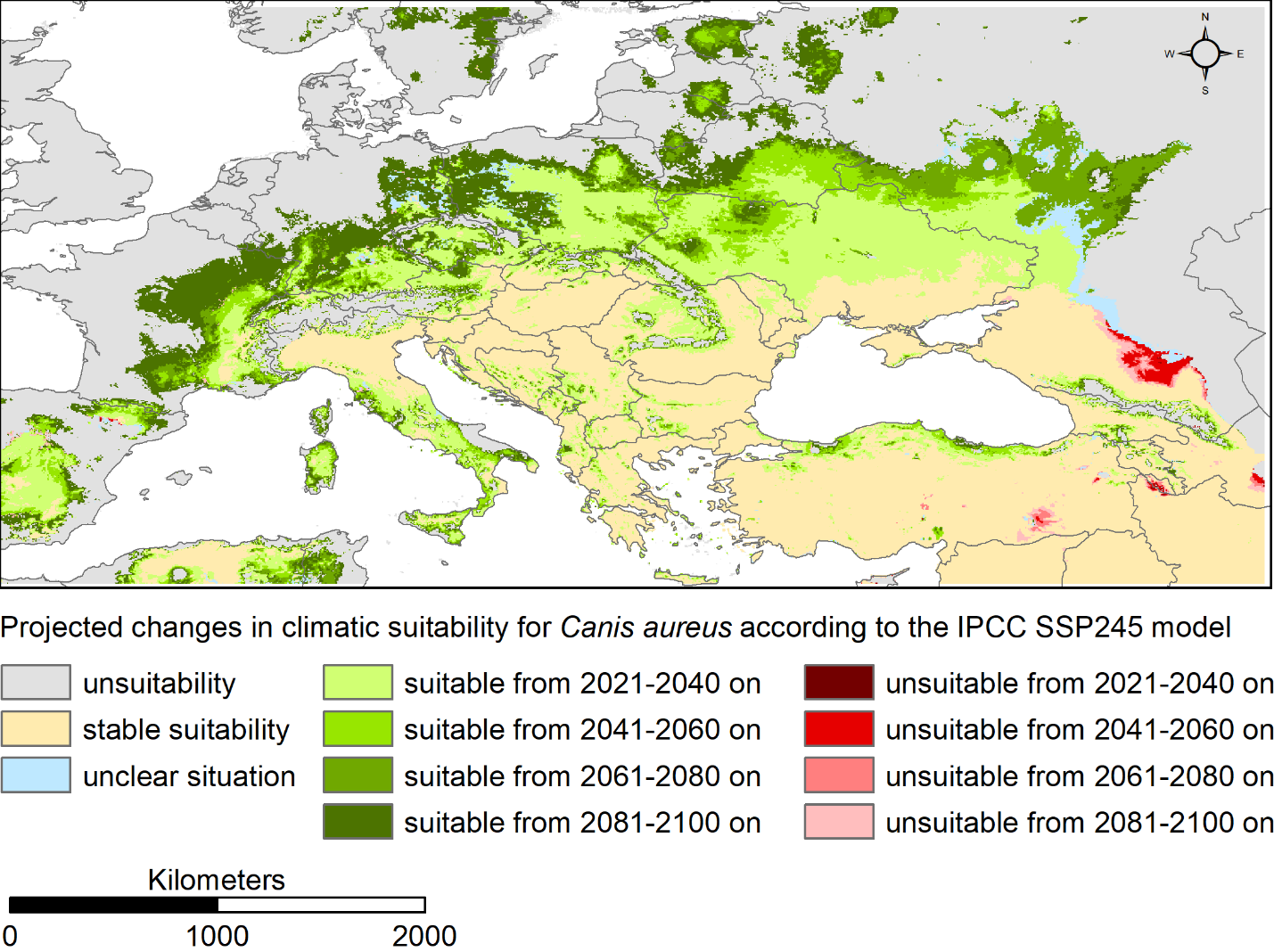


Figure S10: Projected changes (ensemble model) in areas of modelled climatic suitability for the golden jackal (*Canis aureus*) in four time steps to 2100 according to the IPCC scenario SSP 245 in relation to near current conditions (1970 - 2000). We applied the threshold (th=0.48) that minimizes the difference between sensitivity and specificity to transform the continuous modelling results into binary ones. The map was created with ESRI ArcMap version 10.8.1. Coordinate system: WGS 1984


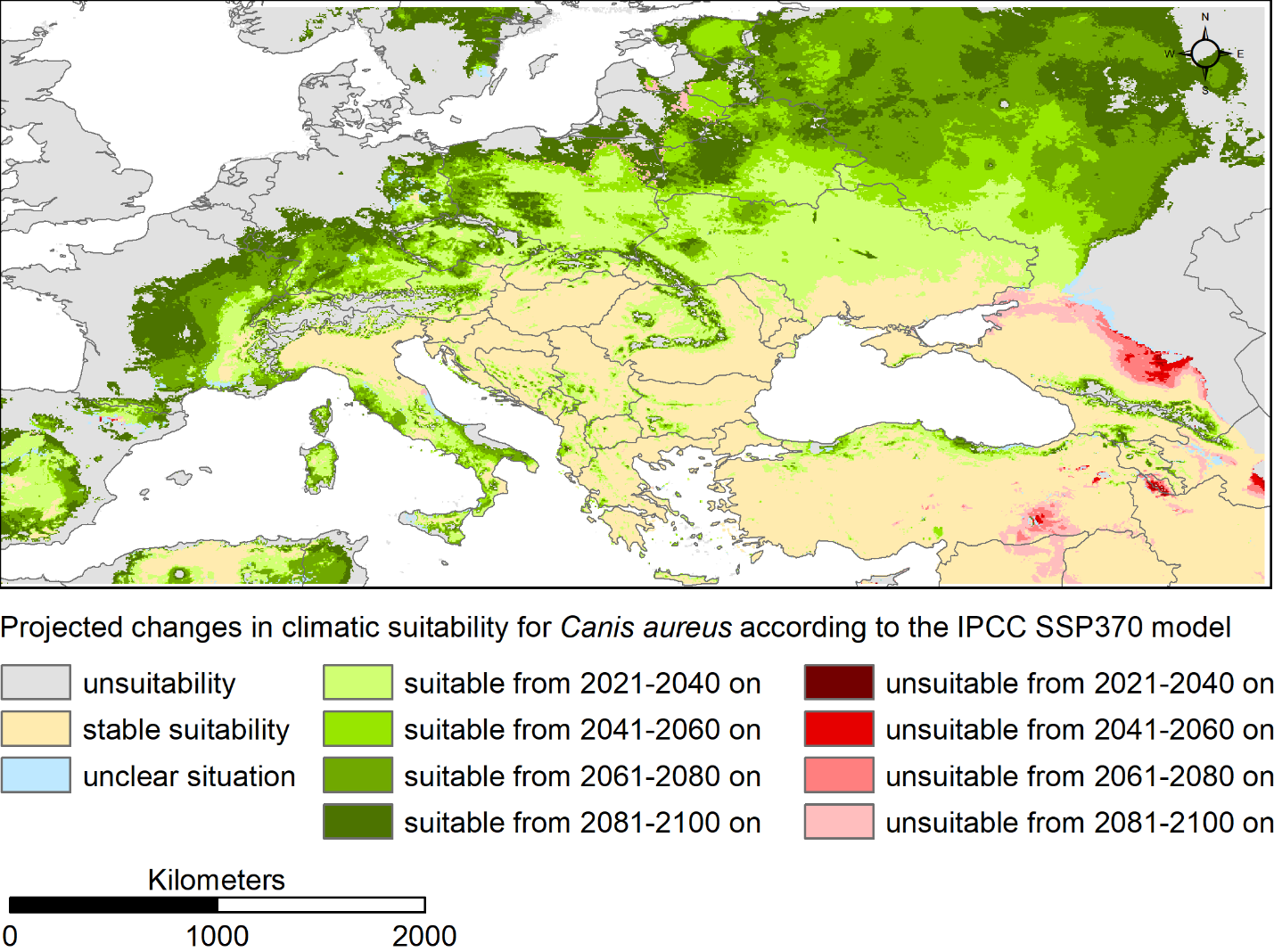


Figure S11: Projected changes (ensemble model) in areas of modelled climatic suitability for the golden jackal (*Canis aureus*) in four time steps to 2100 according to the IPCC scenario SSP 370 in relation to near current conditions (1970 - 2000). We applied the threshold (th=0.48) that minimizes the difference between sensitivity and specificity to transform the continuous modelling results into binary ones. The map was created with ESRI ArcMap version 10.8.1. Coordinate system: WGS 1984


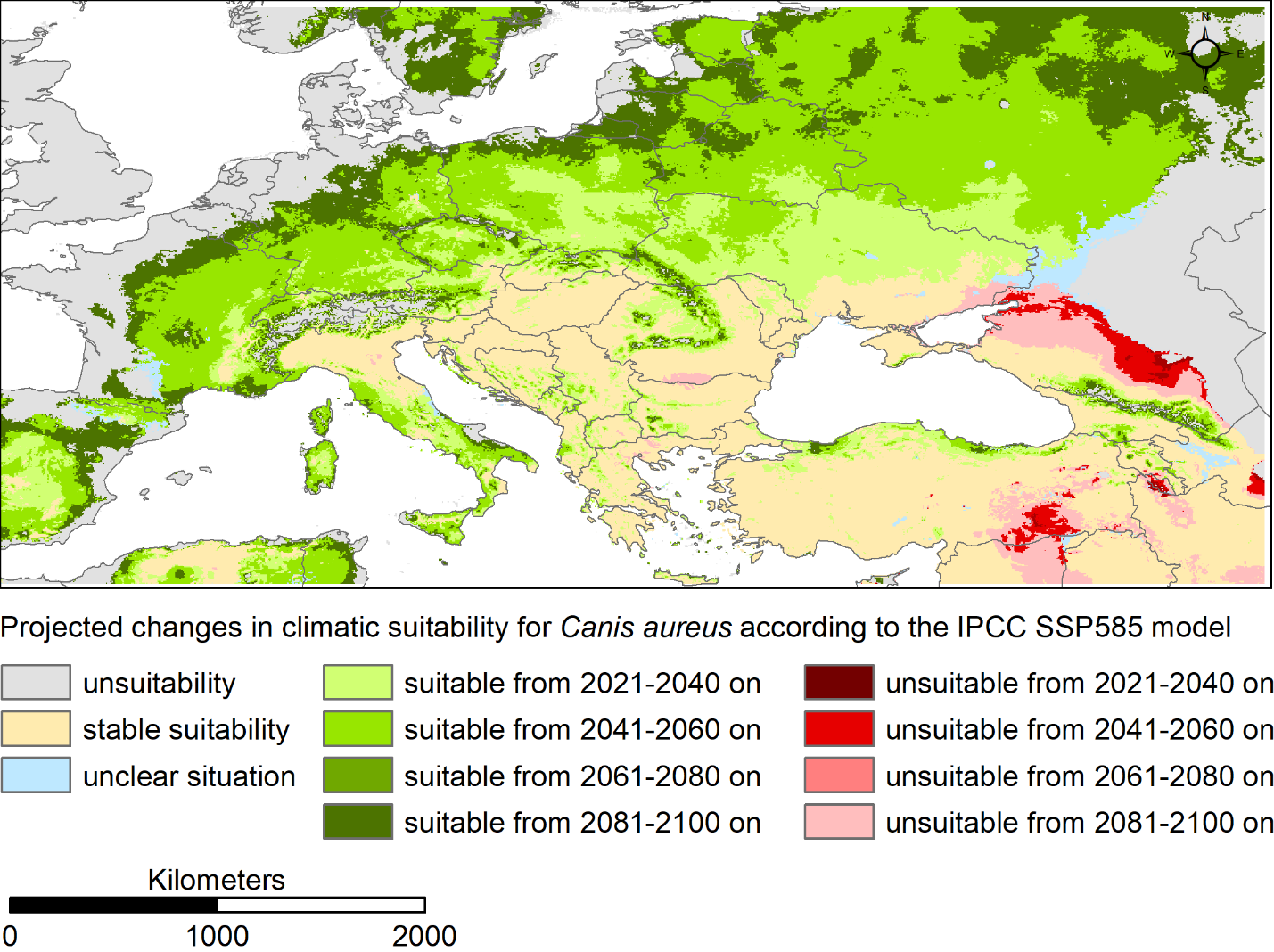


Figure S12: Projected changes (ensemble model) in areas of modelled climatic suitability for the golden jackal (*Canis aureus*) in four time steps to 2100 according to the IPCC scenario SSP 585 in relation to near current conditions (1970 - 2000). We applied the threshold (th=0.48) that minimizes the difference between sensitivity and specificity to transform the continuous modelling results into binary ones. The map was created with ESRI ArcMap version 10.8.1. Coordinate system: WGS 1984


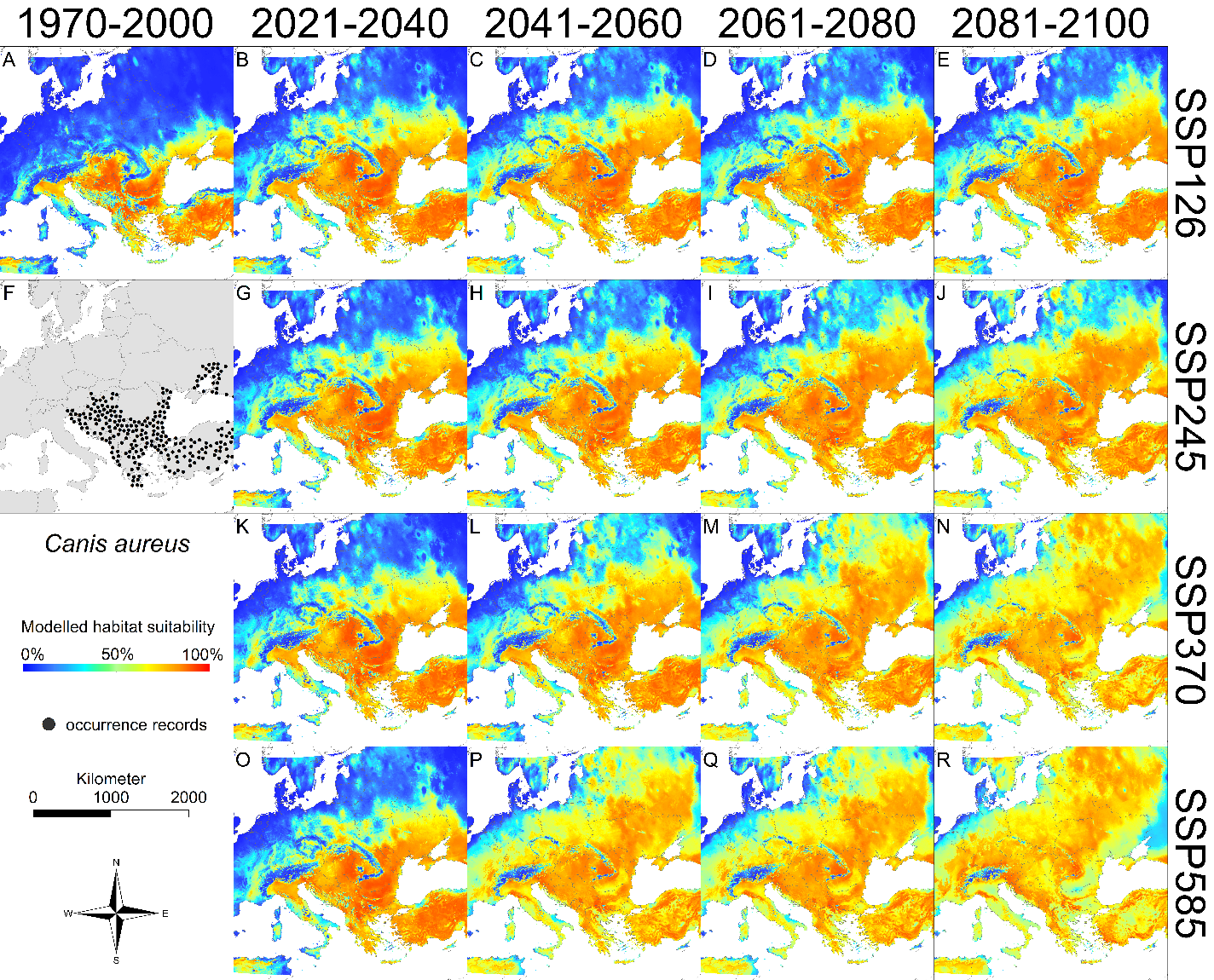


Figure S13: Projected climatic suitability for the golden jackal (*Canis aureus*) under near current climatic conditions and projected future conditions considering four time periods and four IPCC scenario SSPs. The map was created with ESRI ArcMap version 10.8.1. Coordinate system: GCS European 1950 (Europe Albers Equal Area Conic)

# Model evaluation

Table S2: Model evaluation. Modelling performance measures Kappa, true skill statistic (TSS) and area under the receiver operating characteristic curve (AUC) for the six considered single algorithms (GLM – generalized linear models, GBM – generalized boosted models, GAM – generalized additive models, ANN – artificial neuronal networks, FDA – Flexible Discriminant Analysis and RF – Random Forest) and the ensemble forecasting consensus model (EF).

|  | **Kappa** | **TSS** | **AUC** |
| --- | --- | --- | --- |
| GLM | 0.409 | 0.756 | 0.936 |
| GBM | 0.533 | 0.788 | 0.956 |
| GAM | 0.463 | 0.801 | 0.949 |
| ANN | 0.448 | 0.852 | 0.949 |
| FDA | 0.429 | 0.757 | 0.935 |
| RF | 0.772 | 0.894 | 0.985 |
| **EF** | **0.656** | **0.858** | **0.977** |

Figure S14: Relative variable contribution to single models. GLM – generalized linear models, GBM – generalized boosted models, GAM – generalized additive models, ANN – artificial neuronal networks, FDA – Flexible Discriminant Analysis and RF – Random Forest

References

Chapron G, Kaczensky P, Linnell JDC, Arx M von, Huber D, Andrén H et al. (2014) Recovery of large carnivores in Europe's modern human-dominated landscapes. *Science (New York, N.Y.)* 346: 1517–1519.

Gherman CM, Mihalca AD (2017) A synoptic overview of golden jackal parasites reveals high diversity of species. *Parasites & vectors* 10: 419.

Grichik VV, Prakapchuk VV, Grebenchuk AE, Rabtsava AA, Tsybovsky IS (2018) Golden jackal (*Canis aureus* L., 1758) - A new species in the theriofauna of Belarus. *Journal of the Belarusian State University. Biology.* 2018: 55–61.

Jirků M, Dostál D, Robovský J, Šálek M (2018) Reproduction of the golden jackal (Canis aureus ) outside current resident breeding populations in Europe: evidence from the Czech Republic. *mammalia* 82: 592–595.

Kowalczyk R, Kołodziej-Sobocińska M, Ruczyńska I, Wójcik JM (2015) Range expansion of the golden jackal (*Canis aureus*) into Poland: first records. *Mammal Research* 60: 411–414.

Kowalczyk R, Wudarczyk M, Wójcik JM, Okarma H (2020) Northernmost record of reproduction of the expanding golden jackal population. *Mammalian Biology* 100: 107–111.

Krofel M, Giannatos G, Ćirovič D, Stoyanov S, Newsome TM (2017) Golden jackal expansion in Europe: a case of mesopredator release triggered by continent-wide wolf persecution? *Hystrix, the Italian Journal of Mammalogy* 28: 9–15.

Männil P, Ranc N (2022) Golden jackal (*Canis aureus*) in Estonia: development of a thriving population in the boreal ecoregion. *Mammal Research* 67: 245–250.

Ranc N, Acosta-Pankov I, Balys V, Bučko J, Cirovic D, Fabijanić N et al. (2022) *Distribution of large carnivores in Europe 2012 - 2016: Distribution map for Golden jackal (Canis aureus)*. Zenodo.

Rykov AM, Kuznetsova AS, Tirronen KF (2022) The first record of the golden jackal (Canis aureus Linnaeus, 1758) in the Russian Subarctic. *Polar Biology* 45: 965–970.

Sobéron J, Peterson AT (2005) Interpretation of models of fundamental ecological niches and species’ distributional areas. *Biodiversity Informatics* 2.

Sørensen O, Lindsø L (2021a) The golden jackal *Canis aureus* detected in Norway – management challenges with naturally dispersed species new to the country. *Fauna* 74: 74–87.

Sørensen O, Lindsø L (2021b) The golden jackal *Canis aureus* detected in Norway – management challenges with naturally dispersed species new to the country. *Fauna* 74: 74–87.

Spassov N, Acosta-Pankov I (2019) Dispersal history of the golden jackal (Canis aureus moreoticus Geoffroy, 1835) in Europe and possible causes of its recent population explosion. *Biodiversity Data Journal* 7: e34825.

Szewczyk M, Nowak S, Niedźwiecka N, Hulva P, Špinkytė-Bačkaitienė R, Demjanovičová K et al. (2019) Dynamic range expansion leads to establishment of a new, genetically distinct wolf population in Central Europe. *Scientific reports* 9: 19003.

Zagorodniuk I (2014) Golden jackal (*Canis aureus*) in Ukraine: Modern Expansion and Status of Species. *Proceedings of the National Museum of Natural History* 12: 100–105.
