# Supplementary material for: From the Balkan towards Western Europe: Range expansion of the golden jackal (Canis aureus)—A climatic niche modeling approach
Source: Ecol Evol. 2022 Jul 24;12(7):e9141. doi: 10.1002/ece3.9141 (PMC9309039; doi:10.1002/ece3.9141)
Supplement: Supplementary file 2 — Table S3 [file ECE3-12-e9141-s002.docx]

Table S3: Coordinates (WGS 1984) of the occurrences used for model training. See material and method section of the main document for details.

| species | decimallon | decimallat |
| --- | --- | --- |
| *Canis aureus* | 81.370811 | 6.253102 |
| *Canis aureus* | 80.893178 | 6.443488 |
| *Canis aureus* | 81.731044 | 6.569538 |
| *Canis aureus* | 80.384254 | 8.030449 |
| *Canis aureus* | 80.91279 | 8.157583 |
| *Canis aureus* | 79.918495 | 8.317698 |
| *Canis aureus* | 76.690417 | 9.890809 |
| *Canis aureus* | 76.171866 | 10.535183 |
| *Canis aureus* | 76.066562 | 11.044417 |
| *Canis aureus* | 76.433334 | 11.716667 |
| *Canis aureus* | 75.384938 | 11.944685 |
| *Canis aureus* | 76.612979 | 12.268958 |
| *Canis aureus* | 77.862511 | 12.400767 |
| *Canis aureus* | 75.288413 | 12.726163 |
| *Canis aureus* | 74.855957 | 12.914142 |
| *Canis aureus* | 80.222758 | 12.997145 |
| *Canis aureus* | 73.965968 | 15.219292 |
| *Canis aureus* | 74.541661 | 15.234949 |
| *Canis aureus* | 74.143314 | 15.66444 |
| *Canis aureus* | 73.443395 | 16.233189 |
| *Canis aureus* | 73.291278 | 16.946596 |
| *Canis aureus* | 79.545592 | 20.613989 |
| *Canis aureus* | 70.833484 | 21.16657 |
| *Canis aureus* | 71.490382 | 21.561271 |
| *Canis aureus* | 79.232279 | 21.635913 |
| *Canis aureus* | 82.9 | 21.76667 |
| *Canis aureus* | 79.441575 | 22.041002 |
| *Canis aureus* | 82.140915 | 22.079655 |
| *Canis aureus* | 69.697784 | 22.212513 |
| *Canis aureus* | 72.212554 | 22.294678 |
| *Canis aureus* | 70.803826 | 22.304318 |
| *Canis aureus* | 80.608063 | 22.411028 |
| *Canis aureus* | 87.300063 | 22.489794 |
| *Canis aureus* | 78.281079 | 23.114533 |
| *Canis aureus* | 71.230545 | 23.147147 |
| *Canis aureus* | 88.594437 | 23.251362 |
| *Canis aureus* | 77.776498 | 23.343434 |
| *Canis aureus* | 86.637502 | 23.524118 |
| *Canis aureus* | 87.340152 | 23.591993 |
| *Canis aureus* | 88.214493 | 23.612074 |
| *Canis aureus* | 81.02928 | 23.689588 |
| *Canis aureus* | 80.189209 | 23.72124 |
| *Canis aureus* | 90.29985 | 23.737637 |
| *Canis aureus* | 91.703861 | 24.3495 |
| *Canis aureus* | 92.290712 | 24.482944 |
| *Canis aureus* | 92.751167 | 24.684954 |
| *Canis aureus* | 80.03755 | 24.750683 |
| *Canis aureus* | 67.757022 | 24.787117 |
| *Canis aureus* | 87.977034 | 24.957884 |
| *Canis aureus* | 85.471266 | 25.271957 |
| *Canis aureus* | 81.403171 | 25.361054 |
| *Canis aureus* | 77.805976 | 25.368062 |
| *Canis aureus* | 76.428005 | 26.004534 |
| *Canis aureus* | 91.700779 | 26.191093 |
| *Canis aureus* | 76.917514 | 26.208444 |
| *Canis aureus* | 80.521156 | 26.551529 |
| *Canis aureus* | 71.299299 | 26.670161 |
| *Canis aureus* | 87.011032 | 26.674458 |
| *Canis aureus* | 78.6412 | 26.7712 |
| *Canis aureus* | 80.983284 | 26.897675 |
| *Canis aureus* | 72.411186 | 27.128279 |
| *Canis aureus* | 56.036263 | 27.16197 |
| *Canis aureus* | 77.49009 | 27.21731 |
| *Canis aureus* | 76.432571 | 27.329075 |
| *Canis aureus* | 84.343098 | 27.561932 |
| *Canis aureus* | 75.408249 | 27.628584 |
| *Canis aureus* | 73.107937 | 27.97516 |
| *Canis aureus* | 54.000206 | 28.122942 |
| *Canis aureus* | 83.946393 | 28.305022 |
| *Canis aureus* | 80.642882 | 28.494794 |
| *Canis aureus* | 81.32388 | 28.540741 |
| *Canis aureus* | 34.96 | 29.58 |
| *Canis aureus* | 78.859979 | 29.583875 |
| *Canis aureus* | 78.185879 | 29.80858 |
| *Canis aureus* | 34.76 | 30.42 |
| *Canis aureus* | 35.22 | 30.66 |
| *Canis aureus* | 34.686137 | 30.958549 |
| *Canis aureus* | 74.943037 | 31.161962 |
| *Canis aureus* | 34.366736 | 31.338373 |
| *Canis aureus* | 65.826278 | 31.500222 |
| *Canis aureus* | 34.87 | 31.54 |
| *Canis aureus* | 35.44 | 31.72 |
| *Canis aureus* | 53.735948 | 31.98124 |
| *Canis aureus* | 34.99 | 32.05 |
| *Canis aureus* | 53.024804 | 32.252617 |
| *Canis aureus* | 48.4 | 32.3022 |
| *Canis aureus* | 34.964575 | 32.578175 |
| *Canis aureus* | 35.553526 | 32.578822 |
| *Canis aureus* | 35.177425 | 33.078843 |
| *Canis aureus* | 35.739494 | 33.199364 |
| *Canis aureus* | 74.07448 | 33.46082 |
| *Canis aureus* | 73 | 33.5 |
| *Canis aureus* | 35.79217 | 33.730336 |
| *Canis aureus* | 73.406234 | 34.064227 |
| *Canis aureus* | 51.519003 | 34.1671 |
| *Canis aureus* | 36.117662 | 34.407074 |
| *Canis aureus* | 45.758006 | 34.944593 |
| *Canis aureus* | 45.362093 | 35.256393 |
| *Canis aureus* | 44.993649 | 36.009811 |
| *Canis aureus* | 50.795405 | 36.182718 |
| *Canis aureus* | 33.928614 | 36.315333 |
| *Canis aureus* | 52.941094 | 36.504919 |
| *Canis aureus* | 55.192782 | 37.112768 |
| *Canis aureus* | 56.224363 | 37.329287 |
| *Canis aureus* | 49.263083 | 37.537972 |
| *Canis aureus* | 55.710672 | 37.646279 |
| *Canis aureus* | 22.158857 | 37.834427 |
| *Canis aureus* | 31.913889 | 38.279167 |
| *Canis aureus* | 23.249833 | 39.181473 |
| *Canis aureus* | 49.184599 | 39.68483 |
| *Canis aureus* | 23.120219 | 40.702125 |
| *Canis aureus* | 26.060106 | 40.784637 |
| *Canis aureus* | 24.706425 | 41.015839 |
| *Canis aureus* | 41.811432 | 41.193607 |
| *Canis aureus* | 45.904742 | 41.285001 |
| *Canis aureus* | 43.255339 | 41.367827 |
| *Canis aureus* | 41.7321 | 41.6869 |
| *Canis aureus* | 26.869577 | 42.175488 |
| *Canis aureus* | 26.176096 | 42.22065 |
| *Canis aureus* | 27.748232 | 42.274609 |
| *Canis aureus* | 23.691747 | 42.436817 |
| *Canis aureus* | 27.870247 | 42.77763 |
| *Canis aureus* | 45.747901 | 42.834436 |
| *Canis aureus* | 26.099522 | 43.594654 |
| *Canis aureus* | 24.855559 | 43.631192 |
| *Canis aureus* | 47.412995 | 43.747554 |
| *Canis aureus* | 28.746866 | 44.452488 |
| *Canis aureus* | 28.101612 | 44.630265 |
| *Canis aureus* | 29.547939 | 45.349591 |
| *Canis aureus* | 40.887422 | 47.092851 |
| *Canis aureus* | 17.9378315 | 47.1949736 |
| *Canis aureus* | 14.8250807 | 45.1491041 |
| *Canis aureus* | 14.9678483 | 45.7201747 |
| *Canis aureus* | 18.0016611 | 45.4108448 |
| *Canis aureus* | 21.1782415 | 41.9725236 |
| *Canis aureus* | 84.2670779 | 26.437336 |
| *Canis aureus* | 72.1050218 | 30.5995977 |
| *Canis aureus* | 44.3879235 | 36.8323753 |
| *Canis aureus* | 78.5108206 | 14.5409671 |
| *Canis aureus* | 49.8089875 | 24.8569856 |
| *Canis aureus* | 57.9419499 | 38.0264726 |
| *Canis aureus* | 84.1603797 | 25.0133429 |
| *Canis aureus* | 66.6777244 | 32.4192071 |
| *Canis aureus* | 59.2671069 | 33.9551398 |
| *Canis aureus* | 53.6501747 | 33.2014584 |
| *Canis aureus* | 77.6802666 | 18.7014219 |
| *Canis aureus* | 74.8194777 | 22.3177987 |
| *Canis aureus* | 55.2610592 | 34.6397196 |
| *Canis aureus* | 56.1233696 | 27.9553039 |
| *Canis aureus* | 65.5375516 | 40.5652058 |
| *Canis aureus* | 52.3017186 | 32.1494884 |
| *Canis aureus* | 61.6044972 | 41.2404059 |
| *Canis aureus* | 65.8982577 | 41.7150956 |
| *Canis aureus* | 75.7977737 | 17.4509313 |
| *Canis aureus* | 61.9740165 | 28.5891069 |
| *Canis aureus* | 95.0638277 | 23.8703546 |
| *Canis aureus* | 67.6814497 | 27.8943072 |
| *Canis aureus* | 78.8865377 | 12.1872937 |
| *Canis aureus* | 66.8819745 | 44.6405743 |
| *Canis aureus* | 18.5001616 | 43.9294415 |
| *Canis aureus* | 94.7838096 | 16.2647864 |
| *Canis aureus* | 71.4281306 | 30.6235002 |
| *Canis aureus* | 101.242498 | 18.209595 |
| *Canis aureus* | 66.4645253 | 43.6303945 |
| *Canis aureus* | 70.9920573 | 39.4353082 |
| *Canis aureus* | 42.4505873 | 37.8428119 |
| *Canis aureus* | 73.6226203 | 29.9348959 |
| *Canis aureus* | 101.225306 | 18.8276669 |
| *Canis aureus* | 64.9180051 | 36.7656935 |
| *Canis aureus* | 85.1483799 | 19.5104359 |
| *Canis aureus* | 70.3052477 | 45.6732746 |
| *Canis aureus* | 81.1966969 | 16.2645469 |
| *Canis aureus* | 105.308238 | 15.0117542 |
| *Canis aureus* | 60.7693196 | 26.5703957 |
| *Canis aureus* | 66.5205501 | 31.3987454 |
| *Canis aureus* | 87.891468 | 23.9829592 |
| *Canis aureus* | 50.8422824 | 24.4101819 |
| *Canis aureus* | 75.1598786 | 14.0397144 |
| *Canis aureus* | 101.98852 | 14.6769715 |
| *Canis aureus* | 58.4012492 | 34.8255783 |
| *Canis aureus* | 81.7443033 | 18.3630096 |
| *Canis aureus* | 106.305204 | 15.5047723 |
| *Canis aureus* | 70.2361749 | 44.7896675 |
| *Canis aureus* | 80.7094148 | 25.1275685 |
| *Canis aureus* | 57.6626145 | 36.6188543 |
| *Canis aureus* | 57.789554 | 38.5698059 |
| *Canis aureus* | 35.4351993 | 36.802291 |
| *Canis aureus* | 71.2490309 | 28.8043044 |
| *Canis aureus* | 40.3404426 | 40.4449674 |
| *Canis aureus* | 37.8930379 | 45.8789247 |
| *Canis aureus* | 69.971535 | 21.2735448 |
| *Canis aureus* | 100.033052 | 19.6445816 |
| *Canis aureus* | 76.8181046 | 19.5002414 |
| *Canis aureus* | 35.5385133 | 31.2249521 |
| *Canis aureus* | 43.4489297 | 38.6778165 |
| *Canis aureus* | 50.2364733 | 36.5164559 |
| *Canis aureus* | 60.8487934 | 37.663382 |
| *Canis aureus* | 83.3144038 | 28.2019658 |
| *Canis aureus* | 71.8333513 | 29.4148388 |
| *Canis aureus* | 64.8535971 | 39.9508362 |
| *Canis aureus* | 71.8639251 | 42.7433049 |
| *Canis aureus* | 36.1064789 | 39.9175157 |
| *Canis aureus* | 54.8465361 | 32.2984293 |
| *Canis aureus* | 79.8277217 | 14.9088053 |
| *Canis aureus* | 47.6361232 | 26.5134005 |
| *Canis aureus* | 64.9676614 | 42.501908 |
| *Canis aureus* | 43.4327076 | 36.7461933 |
| *Canis aureus* | 38.5403595 | 38.1106304 |
| *Canis aureus* | 64.0790345 | 36.0900076 |
| *Canis aureus* | 64.963066 | 26.7430021 |
| *Canis aureus* | 64.4115477 | 46.0725633 |
| *Canis aureus* | 58.0791839 | 28.3311499 |
| *Canis aureus* | 102.08957 | 16.3319373 |
| *Canis aureus* | 30.5781316 | 46.4878704 |
| *Canis aureus* | 73.3416738 | 19.5263649 |
| *Canis aureus* | 65.475213 | 32.9558169 |
| *Canis aureus* | 50.673234 | 30.9479906 |
| *Canis aureus* | 95.788705 | 20.9854727 |
| *Canis aureus* | 62.0166045 | 40.8304124 |
| *Canis aureus* | 63.0631455 | 27.8689228 |
| *Canis aureus* | 75.832363 | 30.6026765 |
| *Canis aureus* | 104.679079 | 14.2274009 |
| *Canis aureus* | 79.9418587 | 16.3939785 |
| *Canis aureus* | 57.8272068 | 29.3366684 |
| *Canis aureus* | 42.6978165 | 35.2447305 |
| *Canis aureus* | 54.0809485 | 34.5926384 |
| *Canis aureus* | 60.7854444 | 40.5548883 |
| *Canis aureus* | 63.0091391 | 36.8351196 |
| *Canis aureus* | 81.9873348 | 19.348329 |
| *Canis aureus* | 76.4286373 | 14.4469692 |
| *Canis aureus* | 65.4385021 | 28.0244736 |
| *Canis aureus* | 57.7840769 | 34.0262362 |
| *Canis aureus* | 67.9678827 | 26.907909 |
| *Canis aureus* | 49.2639835 | 33.1105955 |
| *Canis aureus* | 73.477787 | 31.3963958 |
| *Canis aureus* | 70.6366752 | 40.0600557 |
| *Canis aureus* | 29.9627559 | 38.8921218 |
| *Canis aureus* | 57.2278357 | 29.298553 |
| *Canis aureus* | 37.0876216 | 38.7767891 |
| *Canis aureus* | 52.2288475 | 36.5452931 |
| *Canis aureus* | 28.7853139 | 38.4572875 |
| *Canis aureus* | 67.0906356 | 40.9371708 |
| *Canis aureus* | 68.9520875 | 39.4687747 |
| *Canis aureus* | 22.655486 | 39.4766493 |
| *Canis aureus* | 103.074649 | 15.0980143 |
| *Canis aureus* | 48.5760104 | 25.8519916 |
| *Canis aureus* | 91.9388599 | 23.933351 |
| *Canis aureus* | 108.196632 | 12.6415631 |
| *Canis aureus* | 94.703606 | 19.0065637 |
| *Canis aureus* | 56.5408742 | 34.6587331 |
| *Canis aureus* | 58.8253369 | 30.7950091 |
| *Canis aureus* | 69.9913695 | 25.0889944 |
| *Canis aureus* | 68.9480847 | 27.8554106 |
| *Canis aureus* | 76.2939097 | 22.5729224 |
| *Canis aureus* | 98.7395132 | 17.3793288 |
| *Canis aureus* | 60.4823949 | 26.0502088 |
| *Canis aureus* | 71.9761486 | 33.5323882 |
| *Canis aureus* | 47.3857136 | 29.2381211 |
| *Canis aureus* | 75.7505694 | 22.7580006 |
| *Canis aureus* | 74.9050568 | 20.5527342 |
| *Canis aureus* | 43.078885 | 37.7429258 |
| *Canis aureus* | 57.8984728 | 39.0275841 |
| *Canis aureus* | 79.489062 | 27.8569847 |
| *Canis aureus* | 66.0262523 | 32.9981969 |
| *Canis aureus* | 73.311073 | 25.5927198 |
| *Canis aureus* | 52.5490923 | 35.2773451 |
| *Canis aureus* | 67.5426855 | 32.6345022 |
| *Canis aureus* | 73.3669698 | 29.3100011 |
| *Canis aureus* | 60.5897768 | 29.4130423 |
| *Canis aureus* | 89.2044905 | 23.6544724 |
| *Canis aureus* | 56.1880849 | 38.6370996 |
| *Canis aureus* | 59.3505232 | 27.7555436 |
| *Canis aureus* | 41.9067874 | 36.0311349 |
| *Canis aureus* | 74.4678762 | 16.4317029 |
| *Canis aureus* | 73.890173 | 17.8297901 |
| *Canis aureus* | 69.1654111 | 35.8560868 |
| *Canis aureus* | 78.6890563 | 25.9111117 |
| *Canis aureus* | 67.0352303 | 30.2667056 |
| *Canis aureus* | 56.779803 | 27.9429436 |
| *Canis aureus* | 75.1402896 | 29.8328714 |
| *Canis aureus* | 63.5841118 | 34.5506491 |
| *Canis aureus* | 83.483004 | 21.4046509 |
| *Canis aureus* | 65.7352883 | 43.4665947 |
| *Canis aureus* | 20.3501136 | 41.0473678 |
| *Canis aureus* | 59.0258175 | 37.4162341 |
| *Canis aureus* | 31.1400095 | 38.6470486 |
| *Canis aureus* | 90.7470418 | 26.1686064 |
| *Canis aureus* | 62.4372336 | 42.4839665 |
| *Canis aureus* | 98.1395237 | 20.2821681 |
| *Canis aureus* | 59.1210878 | 30.1479479 |
| *Canis aureus* | 40.2251594 | 36.1463088 |
| *Canis aureus* | 71.5371589 | 43.7320357 |
| *Canis aureus* | 32.7741824 | 36.8142432 |
| *Canis aureus* | 81.2367635 | 20.5391243 |
| *Canis aureus* | 43.6391 | 37.3948831 |
| *Canis aureus* | 47.6628241 | 42.2950811 |
| *Canis aureus* | 94.313199 | 24.9076315 |
| *Canis aureus* | 46.9375409 | 40.5174947 |
| *Canis aureus* | 84.8504893 | 25.1455634 |
| *Canis aureus* | 69.7245141 | 25.9549591 |
| *Canis aureus* | 63.2254626 | 31.9496377 |
| *Canis aureus* | 35.6879315 | 39.178184 |
| *Canis aureus* | 19.2396226 | 47.4499217 |
| *Canis aureus* | 76.9043978 | 29.918997 |
| *Canis aureus* | 57.0893205 | 41.3935533 |
| *Canis aureus* | 77.0268254 | 18.4857779 |
| *Canis aureus* | 77.4785279 | 29.2156293 |
| *Canis aureus* | 75.9051726 | 11.8696738 |
| *Canis aureus* | 73.7634437 | 25.9353017 |
| *Canis aureus* | 82.6041858 | 20.6224015 |
| *Canis aureus* | 52.0157659 | 31.580349 |
| *Canis aureus* | 76.9938578 | 30.9200683 |
| *Canis aureus* | 95.6336131 | 21.7721267 |
| *Canis aureus* | 56.9793527 | 32.5223237 |
| *Canis aureus* | 50.2880963 | 34.5251238 |
| *Canis aureus* | 60.5353958 | 42.8984927 |
| *Canis aureus* | 55.2747212 | 39.9359431 |
| *Canis aureus* | 65.675219 | 29.2408186 |
| *Canis aureus* | 60.0344132 | 35.6642319 |
| *Canis aureus* | 69.4580738 | 38.0646852 |
| *Canis aureus* | 65.5901208 | 42.2776874 |
| *Canis aureus* | 84.3025593 | 23.2437158 |
| *Canis aureus* | 38.0413511 | 36.9840606 |
| *Canis aureus* | 107.512362 | 12.1609221 |
| *Canis aureus* | 63.5085207 | 26.9389509 |
| *Canis aureus* | 59.8789047 | 39.4564203 |
| *Canis aureus* | 22.1650497 | 40.8397697 |
| *Canis aureus* | 23.1970154 | 41.964889 |
| *Canis aureus* | 86.0330566 | 23.5319034 |
| *Canis aureus* | 91.1025865 | 23.576839 |
| *Canis aureus* | 53.4812211 | 27.0637304 |
| *Canis aureus* | 84.0781059 | 26.0026773 |
| *Canis aureus* | 70.1530729 | 30.2391298 |
| *Canis aureus* | 55.781705 | 35.2581944 |
| *Canis aureus* | 68.0644357 | 28.5697896 |
| *Canis aureus* | 82.0991194 | 20.1750159 |
| *Canis aureus* | 19.2045653 | 45.1162376 |
| *Canis aureus* | 84.7101021 | 23.9050159 |
| *Canis aureus* | 61.9749866 | 37.0768182 |
| *Canis aureus* | 71.2630085 | 41.7188618 |
| *Canis aureus* | 61.818498 | 33.8055193 |
| *Canis aureus* | 78.2674794 | 10.8444108 |
| *Canis aureus* | 74.5471492 | 30.3710852 |
| *Canis aureus* | 46.9667259 | 41.8318894 |
| *Canis aureus* | 48.26833 | 35.2367618 |
| *Canis aureus* | 59.2705701 | 42.6832811 |
| *Canis aureus* | 99.0065655 | 19.642266 |
| *Canis aureus* | 72.5096347 | 28.5493005 |
| *Canis aureus* | 78.38737 | 11.8372986 |
| *Canis aureus* | 70.2095321 | 28.3225326 |
| *Canis aureus* | 82.397774 | 25.5405744 |
| *Canis aureus* | 41.738292 | 35.4139606 |
| *Canis aureus* | 91.7599847 | 25.4104193 |
| *Canis aureus* | 93.7100588 | 22.0893696 |
| *Canis aureus* | 73.9795616 | 20.6780758 |
| *Canis aureus* | 63.6561114 | 37.9360597 |
| *Canis aureus* | 38.730202 | 36.1611322 |
| *Canis aureus* | 94.8421221 | 24.5744255 |
| *Canis aureus* | 39.9875923 | 38.1044404 |
| *Canis aureus* | 43.9257104 | 34.7431786 |
| *Canis aureus* | 88.4990642 | 24.5035657 |
| *Canis aureus* | 81.8429994 | 17.8307132 |
| *Canis aureus* | 62.364468 | 25.8488859 |
| *Canis aureus* | 92.4342808 | 21.1513563 |
| *Canis aureus* | 51.3348535 | 30.0854987 |
| *Canis aureus* | 59.9344987 | 31.062837 |
| *Canis aureus* | 57.0300916 | 42.300301 |
| *Canis aureus* | 68.2826311 | 42.1695633 |
| *Canis aureus* | 77.1640134 | 21.8089618 |
| *Canis aureus* | 79.239092 | 25.5448326 |
| *Canis aureus* | 74.235076 | 22.280322 |
| *Canis aureus* | 48.0221982 | 39.0198113 |
| *Canis aureus* | 62.6211552 | 41.9442796 |
| *Canis aureus* | 55.275012 | 33.915615 |
| *Canis aureus* | 78.9777659 | 19.8805019 |
| *Canis aureus* | 43.8618641 | 43.4410818 |
| *Canis aureus* | 37.5173626 | 39.2667158 |
| *Canis aureus* | 36.9357151 | 35.6874176 |
| *Canis aureus* | 81.7795246 | 28.8837637 |
| *Canis aureus* | 42.2211592 | 34.571557 |
| *Canis aureus* | 59.8330214 | 38.3804888 |
| *Canis aureus* | 73.6993407 | 27.7488975 |
| *Canis aureus* | 58.8088415 | 36.8261351 |
| *Canis aureus* | 77.7672705 | 16.6867612 |
| *Canis aureus* | 62.0293578 | 44.0070835 |
| *Canis aureus* | 63.5388424 | 33.8960585 |
| *Canis aureus* | 45.9140693 | 36.9971851 |
| *Canis aureus* | 58.5637584 | 32.2404766 |
| *Canis aureus* | 49.8382829 | 30.971931 |
| *Canis aureus* | 102.623746 | 17.3355124 |
| *Canis aureus* | 94.7519368 | 21.0676287 |
| *Canis aureus* | 53.0949212 | 35.9583982 |
| *Canis aureus* | 63.6458884 | 28.5077655 |
| *Canis aureus* | 48.5465664 | 38.0918985 |
| *Canis aureus* | 48.2622176 | 31.8223271 |
| *Canis aureus* | 101.879641 | 18.8556852 |
| *Canis aureus* | 50.4345368 | 32.8413825 |
| *Canis aureus* | 79.505616 | 19.9001109 |
| *Canis aureus* | 22.5788201 | 40.0604398 |
| *Canis aureus* | 58.3754359 | 29.6854751 |
| *Canis aureus* | 59.3387451 | 42.0842418 |
| *Canis aureus* | 47.6353062 | 37.6074633 |
| *Canis aureus* | 59.1807534 | 36.0217706 |
| *Canis aureus* | 69.0843541 | 36.3124635 |
| *Canis aureus* | 99.3630357 | 14.362408 |
| *Canis aureus* | 40.542963 | 39.1021186 |
| *Canis aureus* | 81.0282451 | 17.5180867 |
| *Canis aureus* | 48.4922697 | 39.3704972 |
| *Canis aureus* | 36.1957696 | 32.8586101 |
| *Canis aureus* | 76.4894177 | 13.5878325 |
| *Canis aureus* | 74.8931987 | 24.8219231 |
| *Canis aureus* | 71.5295069 | 25.5833929 |
| *Canis aureus* | 64.164141 | 42.5759933 |
| *Canis aureus* | 95.4433221 | 17.0238889 |
| *Canis aureus* | 85.5346847 | 26.9756556 |
| *Canis aureus* | 78.4333128 | 13.8496855 |
| *Canis aureus* | 87.7952677 | 22.361216 |
| *Canis aureus* | 58.9650067 | 32.8972408 |
| *Canis aureus* | 59.7394097 | 36.4570595 |
| *Canis aureus* | 49.4451929 | 34.516771 |
| *Canis aureus* | 56.1036764 | 30.3997546 |
| *Canis aureus* | 44.5764522 | 43.5633001 |
| *Canis aureus* | 21.5249083 | 40.6918202 |
| *Canis aureus* | 82.569941 | 22.8920309 |
| *Canis aureus* | 63.0625258 | 36.0371346 |
| *Canis aureus* | 85.2152767 | 24.4526174 |
| *Canis aureus* | 76.1865903 | 26.4222203 |
| *Canis aureus* | 98.375207 | 18.8905143 |
| *Canis aureus* | 51.1808477 | 30.7196096 |
| *Canis aureus* | 47.9215466 | 40.9103051 |
| *Canis aureus* | 96.605609 | 22.5825363 |
| *Canis aureus* | 58.9570123 | 34.8136708 |
| *Canis aureus* | 75.3340992 | 25.3524767 |
| *Canis aureus* | 56.4576846 | 33.9028182 |
| *Canis aureus* | 78.2137332 | 16.3722267 |
| *Canis aureus* | 67.0445957 | 27.6425567 |
| *Canis aureus* | 20.349541 | 40.5008435 |
| *Canis aureus* | 47.7304636 | 30.4873845 |
| *Canis aureus* | 60.6741321 | 38.6680412 |
| *Canis aureus* | 51.8873707 | 32.6991066 |
| *Canis aureus* | 52.7649723 | 29.5501689 |
| *Canis aureus* | 101.851211 | 14.2412902 |
| *Canis aureus* | 80.0738839 | 27.8550362 |
| *Canis aureus* | 66.9136095 | 33.1590999 |
| *Canis aureus* | 65.8376028 | 25.9570782 |
| *Canis aureus* | 65.5078163 | 37.6007381 |
| *Canis aureus* | 67.2945084 | 42.4619965 |
| *Canis aureus* | 61.4607833 | 32.2001141 |
| *Canis aureus* | 59.3116599 | 28.4215807 |
| *Canis aureus* | 65.464051 | 30.3068395 |
| *Canis aureus* | 76.4275967 | 25.2754828 |
| *Canis aureus* | 46.0725328 | 38.5393968 |
| *Canis aureus* | 67.4969562 | 29.1734434 |
| *Canis aureus* | 79.5895074 | 22.9641746 |
| *Canis aureus* | 49.5880376 | 34.9740454 |
| *Canis aureus* | 76.5021681 | 31.1755159 |
| *Canis aureus* | 67.2991741 | 43.9946444 |
| *Canis aureus* | 72.0536744 | 29.9965555 |
| *Canis aureus* | 77.9607188 | 17.4903947 |
| *Canis aureus* | 34.1002485 | 39.1512474 |
| *Canis aureus* | 41.8617699 | 36.5808062 |
| *Canis aureus* | 47.8990274 | 33.7185486 |
| *Canis aureus* | 102.561147 | 16.215931 |
| *Canis aureus* | 68.2539876 | 35.1981906 |
| *Canis aureus* | 57.8339051 | 37.5335678 |
| *Canis aureus* | 63.8314545 | 30.1841552 |
| *Canis aureus* | 60.3523857 | 39.1531754 |
| *Canis aureus* | 45.2281931 | 38.1302521 |
| *Canis aureus* | 67.4434101 | 34.1955712 |
| *Canis aureus* | 54.6213563 | 27.8046366 |
| *Canis aureus* | 70.8635957 | 42.6327574 |
| *Canis aureus* | 43.202606 | 34.328612 |
| *Canis aureus* | 79.3639581 | 29.1940051 |
| *Canis aureus* | 83.7439373 | 26.4645467 |
| *Canis aureus* | 20.8937474 | 43.0145786 |
| *Canis aureus* | 70.6541556 | 41.8196963 |
| *Canis aureus* | 64.6623846 | 45.3415243 |
| *Canis aureus* | 90.1420065 | 26.989235 |
| *Canis aureus* | 94.3676026 | 20.6079066 |
| *Canis aureus* | 80.5793739 | 19.4616878 |
| *Canis aureus* | 99.0923532 | 18.1054871 |
| *Canis aureus* | 97.6268613 | 16.6736042 |
| *Canis aureus* | 64.8298831 | 33.6915462 |
| *Canis aureus* | 60.9896113 | 33.8377863 |
| *Canis aureus* | 57.5295368 | 28.6215065 |
| *Canis aureus* | 74.2588508 | 29.418009 |
| *Canis aureus* | 74.8624064 | 18.800058 |
| *Canis aureus* | 20.0702078 | 42.839608 |
| *Canis aureus* | 75.7388474 | 26.1440881 |
| *Canis aureus* | 51.6025045 | 36.5831388 |
| *Canis aureus* | 55.1297638 | 29.5563995 |
| *Canis aureus* | 64.6690163 | 29.1554887 |
| *Canis aureus* | 38.4063745 | 48.0294971 |
| *Canis aureus* | 97.9414917 | 14.5404434 |
| *Canis aureus* | 46.8571955 | 32.6117457 |
| *Canis aureus* | 76.6262053 | 23.4707067 |
| *Canis aureus* | 59.7683606 | 31.6818122 |
| *Canis aureus* | 63.4651589 | 42.4324091 |
| *Canis aureus* | 69.3738084 | 29.9400407 |
| *Canis aureus* | 98.8101869 | 15.9322627 |
| *Canis aureus* | 75.9078424 | 26.921358 |
| *Canis aureus* | 56.8835924 | 31.6252254 |
| *Canis aureus* | 69.1515347 | 26.7435707 |
| *Canis aureus* | 59.5979418 | 29.3786526 |
| *Canis aureus* | 67.5663957 | 42.9371322 |
| *Canis aureus* | 63.5011785 | 45.2637557 |
| *Canis aureus* | 67.8160469 | 45.6573832 |
| *Canis aureus* | 64.5804716 | 43.7736152 |
| *Canis aureus* | 60.0483353 | 25.7466179 |
| *Canis aureus* | 74.5977344 | 28.1232517 |
| *Canis aureus* | 55.6391884 | 30.7298169 |
| *Canis aureus* | 42.7033507 | 36.6689212 |
| *Canis aureus* | 70.8212464 | 27.0187645 |
| *Canis aureus* | 95.9283531 | 16.4547223 |
| *Canis aureus* | 79.93165 | 18.4961675 |
| *Canis aureus* | 59.9378995 | 26.4146585 |
| *Canis aureus* | 60.4012231 | 39.9932899 |
| *Canis aureus* | 36.4851183 | 37.0880546 |
| *Canis aureus* | 92.0942428 | 22.061633 |
| *Canis aureus* | 21.8947405 | 40.2477552 |
| *Canis aureus* | 62.2631983 | 30.6201544 |
| *Canis aureus* | 94.6152063 | 21.6350059 |
| *Canis aureus* | 67.7578381 | 26.1617178 |
| *Canis aureus* | 43.306221 | 39.1324172 |
| *Canis aureus* | 104.453303 | 15.2650405 |
| *Canis aureus* | 46.3865356 | 37.2790038 |
| *Canis aureus* | 62.3795792 | 27.2199599 |
| *Canis aureus* | 81.8316819 | 16.5414629 |
| *Canis aureus* | 64.5517768 | 34.1559579 |
| *Canis aureus* | 52.9734024 | 29.1207955 |
| *Canis aureus* | 61.4509622 | 26.3363754 |
| *Canis aureus* | 94.7721865 | 17.3431012 |
| *Canis aureus* | 106.980497 | 15.193911 |
| *Canis aureus* | 78.319274 | 18.631308 |
| *Canis aureus* | 76.2229912 | 18.0381188 |
| *Canis aureus* | 78.0176324 | 20.0192469 |
| *Canis aureus* | 48.6820764 | 32.8222872 |
| *Canis aureus* | 65.5992246 | 44.9350221 |
| *Canis aureus* | 98.5989368 | 20.6926352 |
| *Canis aureus* | 71.9428296 | 28.8639012 |
| *Canis aureus* | 106.608082 | 14.3110407 |
| *Canis aureus* | 63.1689745 | 31.3109206 |
| *Canis aureus* | 98.188184 | 16.8116998 |
| *Canis aureus* | 57.3984189 | 26.6487625 |
| *Canis aureus* | 19.4100634 | 42.6867355 |
| *Canis aureus* | 63.5403986 | 37.2657311 |
| *Canis aureus* | 46.0609565 | 30.59649 |
| *Canis aureus* | 74.3700658 | 21.1543948 |
| *Canis aureus* | 54.3052485 | 37.4592327 |
| *Canis aureus* | 62.6823689 | 39.7945904 |
| *Canis aureus* | 68.2887468 | 27.2809335 |
| *Canis aureus* | 35.9475576 | 37.3232699 |
| *Canis aureus* | 92.5784504 | 22.5971287 |
| *Canis aureus* | 68.8496213 | 34.2738236 |
| *Canis aureus* | 81.9975064 | 23.7956851 |
| *Canis aureus* | 65.3638597 | 44.2518999 |
| *Canis aureus* | 32.4573782 | 38.0739035 |
| *Canis aureus* | 71.762341 | 41.2627098 |
| *Canis aureus* | 69.0346318 | 25.0069283 |
| *Canis aureus* | 62.8053334 | 39.3313175 |
| *Canis aureus* | 80.0099908 | 20.3167967 |
| *Canis aureus* | 61.6220259 | 25.6763077 |
| *Canis aureus* | 34.6366123 | 37.4800567 |
| *Canis aureus* | 26.7222088 | 39.9944404 |
| *Canis aureus* | 100.518712 | 15.9645245 |
| *Canis aureus* | 56.0604573 | 39.9168302 |
| *Canis aureus* | 79.2667876 | 16.1152759 |
| *Canis aureus* | 75.6341152 | 14.19442 |
| *Canis aureus* | 104.662148 | 16.6917575 |
| *Canis aureus* | 41.5879883 | 38.2448604 |
| *Canis aureus* | 63.8118407 | 44.8574175 |
| *Canis aureus* | 20.9356735 | 44.0959837 |
| *Canis aureus* | 64.2457262 | 31.7733998 |
| *Canis aureus* | 85.0108139 | 25.9821018 |
| *Canis aureus* | 74.4205821 | 24.2820065 |
| *Canis aureus* | 90.2241935 | 23.1149553 |
| *Canis aureus* | 59.3711559 | 41.5665067 |
| *Canis aureus* | 101.665228 | 16.523278 |
| *Canis aureus* | 98.1215007 | 21.7132215 |
| *Canis aureus* | 84.7534533 | 24.6840254 |
| *Canis aureus* | 75.9201995 | 18.9372325 |
| *Canis aureus* | 45.3905299 | 33.7959436 |
| *Canis aureus* | 65.1986524 | 38.8148466 |
| *Canis aureus* | 44.0404241 | 35.6942508 |
| *Canis aureus* | 80.2867901 | 17.5403531 |
| *Canis aureus* | 69.7539676 | 40.4045482 |
| *Canis aureus* | 67.5169827 | 44.4242264 |
| *Canis aureus* | 80.614704 | 16.7430373 |
| *Canis aureus* | 96.6592064 | 24.2825996 |
| *Canis aureus* | 70.1780077 | 36.9530331 |
| *Canis aureus* | 79.8022474 | 11.0131866 |
| *Canis aureus* | 66.8571736 | 45.9367184 |
| *Canis aureus* | 61.3207991 | 39.5543274 |
| *Canis aureus* | 83.206568 | 17.9736376 |
| *Canis aureus* | 79.6347746 | 11.8961837 |
| *Canis aureus* | 46.6628653 | 29.9338696 |
| *Canis aureus* | 40.7868511 | 41.1236004 |
| *Canis aureus* | 69.4727572 | 45.1271168 |
| *Canis aureus* | 61.6278451 | 29.8588866 |
| *Canis aureus* | 62.1049814 | 32.7782303 |
| *Canis aureus* | 22.0208298 | 38.7197211 |
| *Canis aureus* | 45.1759353 | 36.764725 |
| *Canis aureus* | 83.0488909 | 27.2446216 |
| *Canis aureus* | 58.3997794 | 33.1070697 |
| *Canis aureus* | 37.6740338 | 36.4790773 |
| *Canis aureus* | 71.4774055 | 44.2861473 |
| *Canis aureus* | 103.594707 | 17.6328731 |
| *Canis aureus* | 66.1761645 | 39.9473229 |
| *Canis aureus* | 51.1951534 | 29.2810146 |
| *Canis aureus* | 76.0686355 | 20.3283358 |
| *Canis aureus* | 79.2911168 | 16.5888415 |
| *Canis aureus* | 80.2957463 | 21.5668554 |
| *Canis aureus* | 38.062101 | 45.1491983 |
| *Canis aureus* | 51.6006635 | 33.5395928 |
| *Canis aureus* | 98.3047239 | 18.2383887 |
| *Canis aureus* | 74.8827458 | 23.38091 |
| *Canis aureus* | 79.3585005 | 11.2415555 |
| *Canis aureus* | 71.6300748 | 28.2798088 |
| *Canis aureus* | 74.0285949 | 20.0555347 |
| *Canis aureus* | 45.3676835 | 33.2941907 |
| *Canis aureus* | 72.1127136 | 24.2810895 |
| *Canis aureus* | 72.1414782 | 23.3654544 |
| *Canis aureus* | 48.0641907 | 36.966039 |
| *Canis aureus* | 69.091062 | 36.8065863 |
| *Canis aureus* | 46.9797652 | 28.73699 |
| *Canis aureus* | 65.1623555 | 31.1316732 |
| *Canis aureus* | 65.1361924 | 32.2718266 |
| *Canis aureus* | 60.2188106 | 41.5084793 |
| *Canis aureus* | 75.0815296 | 17.455478 |
| *Canis aureus* | 77.1835802 | 28.7185304 |
| *Canis aureus* | 93.8860534 | 22.9270206 |
| *Canis aureus* | 48.644574 | 36.2887486 |
| *Canis aureus* | 36.5209454 | 40.6107765 |
| *Canis aureus* | 85.7797089 | 23.9681521 |
| *Canis aureus* | 43.8971993 | 37.8956694 |
| *Canis aureus* | 103.957582 | 18.8906791 |
| *Canis aureus* | 88.7229064 | 22.0103308 |
| *Canis aureus* | 56.8914608 | 31.0505887 |
| *Canis aureus* | 37.6941327 | 35.0579021 |
| *Canis aureus* | 56.17936 | 31.2948605 |
| *Canis aureus* | 99.0250766 | 20.4547013 |
| *Canis aureus* | 17.5853222 | 44.5304074 |
| *Canis aureus* | 77.4390906 | 30.257184 |
| *Canis aureus* | 48.1748624 | 28.8220989 |
| *Canis aureus* | 51.4729407 | 28.5056952 |
| *Canis aureus* | 61.0684314 | 36.3387866 |
| *Canis aureus* | 70.0245396 | 24.509203 |
| *Canis aureus* | 52.3649541 | 30.2897385 |
| *Canis aureus* | 62.9029959 | 44.9031079 |
| *Canis aureus* | 78.1749512 | 26.9797046 |
| *Canis aureus* | 100.396945 | 17.4087404 |
| *Canis aureus* | 62.8427188 | 26.7105449 |
| *Canis aureus* | 41.1574009 | 43.4254299 |
| *Canis aureus* | 23.1603285 | 43.0640406 |
| *Canis aureus* | 70.5874381 | 29.4504871 |
| *Canis aureus* | 62.0775995 | 32.0411097 |
| *Canis aureus* | 57.4520087 | 31.1837464 |
| *Canis aureus* | 61.1422042 | 28.2341954 |
| *Canis aureus* | 67.6840224 | 27.3792048 |
| *Canis aureus* | 68.5150673 | 30.8954416 |
| *Canis aureus* | 46.6898014 | 34.0415598 |
| *Canis aureus* | 68.1603626 | 31.2652907 |
| *Canis aureus* | 46.4979141 | 30.8674878 |
| *Canis aureus* | 56.7838651 | 28.6527194 |
| *Canis aureus* | 100.482722 | 19.3879133 |
| *Canis aureus* | 66.9952357 | 37.0973092 |
| *Canis aureus* | 41.5929822 | 39.3178735 |
| *Canis aureus* | 63.7054141 | 45.7219832 |
| *Canis aureus* | 76.065913 | 29.1918037 |
| *Canis aureus* | 56.1390739 | 42.0205211 |
| *Canis aureus* | 67.0677219 | 35.312683 |
| *Canis aureus* | 76.1529032 | 21.9797403 |
| *Canis aureus* | 76.8181381 | 15.4210647 |
| *Canis aureus* | 76.7968403 | 19.9665179 |
| *Canis aureus* | 68.0936009 | 40.2589426 |
| *Canis aureus* | 92.9931197 | 25.309454 |
| *Canis aureus* | 74.6099533 | 32.829482 |
| *Canis aureus* | 60.3149839 | 31.8290531 |
| *Canis aureus* | 74.2167816 | 18.617085 |
| *Canis aureus* | 54.8274242 | 35.9106665 |
| *Canis aureus* | 15.7677853 | 44.6665748 |
| *Canis aureus* | 66.4855182 | 33.9848536 |
| *Canis aureus* | 53.865735 | 29.6451938 |
| *Canis aureus* | 78.775727 | 24.2243888 |
| *Canis aureus* | 47.1384861 | 35.1416956 |
| *Canis aureus* | 73.2559092 | 17.6197969 |
| *Canis aureus* | 45.0416631 | 34.5419062 |
| *Canis aureus* | 75.5487157 | 15.7562041 |
| *Canis aureus* | 83.8947495 | 22.5005196 |
| *Canis aureus* | 80.6053086 | 24.5235453 |
| *Canis aureus* | 75.2593817 | 15.1241913 |
| *Canis aureus* | 97.8042592 | 17.2103555 |
| *Canis aureus* | 67.7927676 | 35.5816467 |
| *Canis aureus* | 81.4735854 | 22.2299157 |
| *Canis aureus* | 88.6233791 | 24.0522648 |
| *Canis aureus* | 48.7408645 | 37.1122687 |
| *Canis aureus* | 61.4265766 | 25.1429193 |
| *Canis aureus* | 89.9558729 | 25.3673343 |
| *Canis aureus* | 76.6997784 | 11.0483292 |
| *Canis aureus* | 97.007082 | 20.787794 |
| *Canis aureus* | 76.7595905 | 10.5640164 |
| *Canis aureus* | 76.1903352 | 24.1660664 |
| *Canis aureus* | 64.2896351 | 38.0540465 |
| *Canis aureus* | 76.2273855 | 15.0170084 |
| *Canis aureus* | 55.3822782 | 39.110407 |
| *Canis aureus* | 45.8331119 | 36.4210458 |
| *Canis aureus* | 84.90849 | 20.7918083 |
| *Canis aureus* | 89.1560877 | 24.6472276 |
| *Canis aureus* | 66.6513568 | 26.5488824 |
| *Canis aureus* | 102.400704 | 17.787306 |
| *Canis aureus* | 103.265708 | 18.8216871 |
| *Canis aureus* | 84.5978112 | 22.0451641 |
| *Canis aureus* | 61.816456 | 31.3260047 |
| *Canis aureus* | 55.1576378 | 38.6479171 |
| *Canis aureus* | 81.2901489 | 27.7705653 |
| *Canis aureus* | 34.6395477 | 46.1226671 |
| *Canis aureus* | 72.1492226 | 40.7460586 |
| *Canis aureus* | 38.6639177 | 38.8248774 |
| *Canis aureus* | 59.1047795 | 25.4689143 |
| *Canis aureus* | 103.229891 | 17.1557466 |
| *Canis aureus* | 45.8279788 | 35.6032744 |
| *Canis aureus* | 85.9843409 | 25.3483759 |
| *Canis aureus* | 94.6360111 | 25.7859525 |
| *Canis aureus* | 78.2434274 | 11.3356762 |
| *Canis aureus* | 50.3722064 | 35.4416289 |
| *Canis aureus* | 107.401676 | 13.6035321 |
| *Canis aureus* | 99.8875778 | 15.9557696 |
| *Canis aureus* | 80.3149756 | 25.5766729 |
| *Canis aureus* | 36.1477091 | 35.5294819 |
| *Canis aureus* | 83.1970372 | 24.6274151 |
| *Canis aureus* | 81.8553393 | 17.0342949 |
| *Canis aureus* | 75.8598339 | 31.5680533 |
| *Canis aureus* | 98.8767181 | 19.1070249 |
| *Canis aureus* | 32.1645777 | 38.89945 |
| *Canis aureus* | 98.6428781 | 20.1737475 |
| *Canis aureus* | 101.504208 | 17.075514 |
| *Canis aureus* | 70.6716068 | 38.9161349 |
| *Canis aureus* | 59.938906 | 29.8560185 |
| *Canis aureus* | 63.8947284 | 32.130774 |
| *Canis aureus* | 69.5867888 | 44.2068237 |
| *Canis aureus* | 94.851934 | 23.0926798 |
| *Canis aureus* | 63.5977262 | 43.2436073 |
| *Canis aureus* | 99.776664 | 19.174003 |
| *Canis aureus* | 61.462424 | 41.6829758 |
| *Canis aureus* | 40.8788558 | 36.2281018 |
| *Canis aureus* | 82.3287443 | 17.7502681 |
| *Canis aureus* | 22.9744811 | 38.7319696 |
| *Canis aureus* | 77.3621691 | 12.6963705 |
| *Canis aureus* | 50.1139152 | 25.5180102 |
| *Canis aureus* | 78.9576258 | 13.7663396 |
| *Canis aureus* | 76.0883935 | 32.061104 |
| *Canis aureus* | 63.5433292 | 35.0312606 |
| *Canis aureus* | 78.6398073 | 21.5525577 |
| *Canis aureus* | 92.7393737 | 23.6013832 |
| *Canis aureus* | 80.5759393 | 20.5129435 |
| *Canis aureus* | 59.201713 | 31.4785317 |
| *Canis aureus* | 96.3047074 | 19.5491742 |
| *Canis aureus* | 93.3282651 | 20.5977712 |
| *Canis aureus* | 71.792836 | 27.7268304 |
| *Canis aureus* | 70.5103067 | 33.7174825 |
| *Canis aureus* | 97.367868 | 22.1253917 |
| *Canis aureus* | 19.6216823 | 43.502215 |
| *Canis aureus* | 104.097702 | 16.9922854 |
| *Canis aureus* | 65.9351018 | 38.2747398 |
| *Canis aureus* | 99.3462146 | 17.126069 |
| *Canis aureus* | 79.1322572 | 10.6326464 |
| *Canis aureus* | 19.6888953 | 41.5723297 |
| *Canis aureus* | 93.2053234 | 21.7906923 |
| *Canis aureus* | 74.0883079 | 24.9117181 |
| *Canis aureus* | 65.0243115 | 28.2899871 |
| *Canis aureus* | 95.9057826 | 20.1070931 |
| *Canis aureus* | 75.3700048 | 23.9048946 |
| *Canis aureus* | 63.2699099 | 44.0073916 |
| *Canis aureus* | 68.3180105 | 36.8809567 |
| *Canis aureus* | 70.6356058 | 27.6890424 |
| *Canis aureus* | 71.6707761 | 22.2079534 |
| *Canis aureus* | 62.5416056 | 29.4577699 |
| *Canis aureus* | 98.9398115 | 13.2312534 |
| *Canis aureus* | 66.1599537 | 38.7535746 |
| *Canis aureus* | 68.9693496 | 45.6180404 |
| *Canis aureus* | 79.2648679 | 19.1307659 |
| *Canis aureus* | 65.7221627 | 34.8913959 |
| *Canis aureus* | 80.6252728 | 17.2249013 |
| *Canis aureus* | 76.977941 | 27.9567263 |
| *Canis aureus* | 54.1897462 | 36.2862788 |
| *Canis aureus* | 35.3614587 | 38.6475004 |
| *Canis aureus* | 41.8306976 | 37.8264569 |
| *Canis aureus* | 48.7414703 | 31.4483978 |
| *Canis aureus* | 46.1211199 | 31.7801672 |
| *Canis aureus* | 81.3194625 | 6.90500952 |
| *Canis aureus* | 80.5660372 | 9.23465008 |
| *Canis aureus* | 80.9782902 | 7.47899974 |
| *Canis aureus* | 79.9813737 | 8.84417595 |
| *Canis aureus* | 80.5867251 | 6.03966164 |
| *Canis aureus* | 80.9944034 | 8.74445992 |
| *Canis aureus* | 80.4583329 | 6.56726367 |
| *Canis aureus* | 80.0110043 | 7.36245441 |
| *Canis aureus* | 80.2189928 | 9.79555297 |
| *Canis aureus* | 80.5324547 | 8.72662163 |
| *Canis aureus* | 81.8041105 | 7.27868385 |
| *Canis aureus* | 81.4194978 | 7.80518553 |
| *Canis aureus* | 80.4770678 | 7.11976729 |
| *Canis aureus* | 81.3454361 | 8.3177819 |
| *Canis aureus* | 80.1037633 | 6.13569412 |
| *Canis aureus* | 79.9463456 | 6.91009532 |
| *Canis aureus* | 13.5039964 | 45.8491393 |
| *Canis aureus* | 13.9751064 | 44.9704556 |
| *Canis aureus* | 18.2108874 | 42.7231809 |
| *Canis aureus* | 16.0624663 | 43.5806121 |
| *Canis aureus* | 14.9372198 | 44.6983769 |
| *Canis aureus* | 17.5643105 | 45.9446274 |
| *Canis aureus* | 19.5575684 | 45.6663679 |
| *Canis aureus* | 20.1568706 | 45.8317058 |
| *Canis aureus* | 28.3981412 | 41.2271403 |
| *Canis aureus* | 27.1501778 | 41.3835693 |
| *Canis aureus* | 28.0263698 | 41.7132494 |
| *Canis aureus* | 26.4134251 | 40.4000074 |
| *Canis aureus* | 29.4438595 | 41.1947093 |
| *Canis aureus* | 27.3213588 | 40.7426277 |
| *Canis aureus* | 27.895941 | 40.9776385 |
| *Canis aureus* | 24.4111477 | 41.9830068 |
| *Canis aureus* | 24.9777104 | 41.4283122 |
| *Canis aureus* | 27.3616767 | 43.5007086 |
| *Canis aureus* | 27.9439043 | 43.3397797 |
| *Canis aureus* | 26.5690618 | 43.2302365 |
| *Canis aureus* | 25.0828792 | 42.0678732 |
| *Canis aureus* | 23.0751345 | 43.7571103 |
| *Canis aureus* | 28.2112543 | 45.5672178 |
| *Canis aureus* | 29.5726821 | 44.8989086 |
| *Canis aureus* | 25.9190295 | 44.2509734 |
| *Canis aureus* | 23.6790435 | 44.1251342 |
| *Canis aureus* | 28.588979 | 43.8594083 |
| *Canis aureus* | 27.2574107 | 44.6788308 |
| *Canis aureus* | 23.3575069 | 40.0709032 |
| *Canis aureus* | 22.928611 | 37.8257063 |
| *Canis aureus* | 21.9532632 | 37.3959226 |
| *Canis aureus* | 12.9819216 | 46.2705296 |
| *Canis aureus* | 13.8212764 | 46.2770422 |
| *Canis aureus* | 14.2867957 | 45.8391157 |
| *Canis aureus* | 13.5709747 | 45.3661738 |
| *Canis aureus* | 15.1752367 | 44.1909993 |
| *Canis aureus* | 16.0127359 | 44.1538166 |
| *Canis aureus* | 17.3960568 | 47.4692164 |
| *Canis aureus* | 17.2067443 | 48.0382633 |
| *Canis aureus* | 20.9275022 | 47.7150488 |
| *Canis aureus* | 20.6618599 | 47.2108711 |
| *Canis aureus* | 21.5822588 | 47.5587969 |
| *Canis aureus* | 18.041708 | 43.3649836 |
| *Canis aureus* | 19.61311 | 40.3877822 |
| *Canis aureus* | 19.441779 | 41.0259221 |
| *Canis aureus* | 19.3315864 | 42.1927965 |
| *Canis aureus* | 17.1496548 | 43.5198772 |
| *Canis aureus* | 18.8232128 | 42.5248801 |
| *Canis aureus* | 25.5807662 | 41.0827969 |
| *Canis aureus* | 26.6336951 | 41.6319569 |
| *Canis aureus* | 26.7045273 | 40.8283683 |
| *Canis aureus* | 27.3643214 | 41.8499624 |
| *Canis aureus* | 17.4705471 | 45.0351602 |
| *Canis aureus* | 19.5979206 | 44.009913 |
| *Canis aureus* | 18.096501 | 44.914983 |
| *Canis aureus* | 16.7082982 | 45.7113743 |
| *Canis aureus* | 15.8647395 | 45.3862251 |
| *Canis aureus* | 16.6622444 | 45.243745 |
| *Canis aureus* | 18.6964532 | 45.492358 |
| *Canis aureus* | 18.0971886 | 46.4339361 |
| *Canis aureus* | 16.9686745 | 46.2835397 |
| *Canis aureus* | 18.3007983 | 45.9336928 |
| *Canis aureus* | 20.5511717 | 46.2328404 |
| *Canis aureus* | 18.864218 | 46.5591818 |
| *Canis aureus* | 19.7365188 | 46.3570609 |
| *Canis aureus* | 17.5376776 | 46.8208233 |
| *Canis aureus* | 19.0799388 | 46.0753245 |
| *Canis aureus* | 19.5445295 | 46.9768568 |
| *Canis aureus* | 18.6327435 | 47.1924174 |
| *Canis aureus* | 21.1761508 | 43.4942456 |
| *Canis aureus* | 20.7943394 | 45.7053856 |
| *Canis aureus* | 19.7983367 | 44.7471593 |
| *Canis aureus* | 20.1481207 | 45.2814219 |
| *Canis aureus* | 20.8900215 | 45.1136443 |
| *Canis aureus* | 21.3066548 | 44.5538432 |
| *Canis aureus* | 21.9634087 | 42.4254548 |
| *Canis aureus* | 21.5786957 | 43.9490705 |
| *Canis aureus* | 19.1572651 | 44.4531381 |
| *Canis aureus* | 21.7987413 | 43.3140418 |
| *Canis aureus* | 22.0580025 | 44.5342916 |
| *Canis aureus* | 22.2744271 | 42.9057757 |
| *Canis aureus* | 20.4764581 | 43.7750097 |
| *Canis aureus* | 20.5620783 | 44.5311972 |
| *Canis aureus* | 22.5232938 | 43.3194996 |
| *Canis aureus* | 15.6455495 | 45.8302787 |
| *Canis aureus* | 16.4350329 | 46.5952714 |
| *Canis aureus* | 24.334234 | 42.5025144 |
| *Canis aureus* | 23.7727171 | 43.1562168 |
| *Canis aureus* | 24.6100799 | 43.0006813 |
| *Canis aureus* | 26.7187325 | 43.8989522 |
| *Canis aureus* | 25.5062679 | 43.32764 |
| *Canis aureus* | 23.8466558 | 43.631996 |
| *Canis aureus* | 25.0778002 | 42.5911906 |
| *Canis aureus* | 26.4081396 | 42.6420294 |
| *Canis aureus* | 22.3744426 | 43.8445992 |
| *Canis aureus* | 25.7271232 | 41.5553295 |
| *Canis aureus* | 27.1441973 | 42.8646777 |
| *Canis aureus* | 29.380841 | 46.635281 |
| *Canis aureus* | 29.4961471 | 47.1447109 |
| *Canis aureus* | 21.2180596 | 46.1861499 |
| *Canis aureus* | 27.1790493 | 44.2262912 |
| *Canis aureus* | 25.373 | 43.9586393 |
| *Canis aureus* | 28.7542848 | 45.1169467 |
| *Canis aureus* | 23.1832953 | 44.5117674 |
| *Canis aureus* | 21.5362982 | 45.0377977 |
| *Canis aureus* | 24.3944483 | 43.9829069 |
| *Canis aureus* | 27.8888173 | 44.1790083 |
| *Canis aureus* | 21.8167891 | 45.5785603 |
| *Canis aureus* | 29.1167927 | 45.7116321 |
| *Canis aureus* | 30.0309501 | 45.88167 |
| *Canis aureus* | 30.1113109 | 46.8232194 |
| *Canis aureus* | 20.3299472 | 39.4828546 |
| *Canis aureus* | 20.1001819 | 39.9326938 |
| *Canis aureus* | 21.4155887 | 38.1727369 |
| *Canis aureus* | 24.0872062 | 40.3098691 |
| *Canis aureus* | 23.9240476 | 40.7763442 |
| *Canis aureus* | 23.2934901 | 41.3803727 |
| *Canis aureus* | 22.6656454 | 41.1457569 |
| *Canis aureus* | 22.6058766 | 41.605634 |
| *Canis aureus* | 24.0357108 | 37.8502444 |
| *Canis aureus* | 29.4180224 | 39.6702692 |
| *Canis aureus* | 29.9403038 | 40.4881731 |
| *Canis aureus* | 31.1047473 | 37.8345732 |
| *Canis aureus* | 35.9152169 | 40.6090693 |
| *Canis aureus* | 30.5436498 | 38.2487794 |
| *Canis aureus* | 35.0694458 | 39.216877 |
| *Canis aureus* | 30.9198841 | 39.0878772 |
| *Canis aureus* | 26.850032 | 38.243079 |
| *Canis aureus* | 42.6539608 | 39.9419199 |
| *Canis aureus* | 34.1849561 | 38.0040647 |
| *Canis aureus* | 33.5760549 | 39.6675219 |
| *Canis aureus* | 29.1908987 | 40.5919483 |
| *Canis aureus* | 38.0073992 | 40.0628742 |
| *Canis aureus* | 33.074174 | 38.5841793 |
| *Canis aureus* | 43.8847826 | 39.5854044 |
| *Canis aureus* | 43.3022844 | 39.8688277 |
| *Canis aureus* | 41.9160967 | 40.6561345 |
| *Canis aureus* | 37.7896895 | 38.484878 |
| *Canis aureus* | 39.3139206 | 39.0356891 |
| *Canis aureus* | 39.9812978 | 39.4000288 |
| *Canis aureus* | 28.50809 | 37.4901686 |
| *Canis aureus* | 34.5949834 | 38.4219196 |
| *Canis aureus* | 39.4916686 | 40.9537158 |
| *Canis aureus* | 28.8502805 | 39.083601 |
| *Canis aureus* | 30.4725595 | 40.7002431 |
| *Canis aureus* | 42.2668903 | 39.2582143 |
| *Canis aureus* | 32.7948092 | 40.2338846 |
| *Canis aureus* | 27.9253585 | 37.2754114 |
| *Canis aureus* | 35.1382694 | 38.2128373 |
| *Canis aureus* | 27.6761989 | 39.5085487 |
| *Canis aureus* | 30.3286848 | 39.9302255 |
| *Canis aureus* | 31.9901908 | 40.1132191 |
| *Canis aureus* | 29.1855444 | 37.1893219 |
| *Canis aureus* | 36.7668325 | 39.6177627 |
| *Canis aureus* | 27.5292372 | 40.2231261 |
| *Canis aureus* | 36.0861386 | 38.1630008 |
| *Canis aureus* | 38.914125 | 40.5833721 |
| *Canis aureus* | 28.4319076 | 39.7395994 |
| *Canis aureus* | 31.5940742 | 40.5069519 |
| *Canis aureus* | 43.2123992 | 40.5103557 |
| *Canis aureus* | 32.7505527 | 39.089474 |
| *Canis aureus* | 38.392795 | 39.4395881 |
| *Canis aureus* | 33.383032 | 39.1847815 |
| *Canis aureus* | 36.1532435 | 38.7843739 |
| *Canis aureus* | 30.3813758 | 37.662983 |
| *Canis aureus* | 39.4999464 | 39.783754 |
| *Canis aureus* | 37.7059745 | 41.0793081 |
| *Canis aureus* | 27.3531398 | 37.6763962 |
| *Canis aureus* | 37.3586535 | 39.7967306 |
| *Canis aureus* | 27.6586702 | 39.0556856 |
| *Canis aureus* | 41.1373578 | 40.3976691 |
| *Canis aureus* | 31.3278655 | 39.8060172 |
| *Canis aureus* | 41.4288238 | 42.8248614 |
| *Canis aureus* | 42.1803548 | 42.0008791 |
| *Canis aureus* | 43.7840598 | 42.2463559 |
| *Canis aureus* | 43.7645053 | 41.649576 |
| *Canis aureus* | 44.7255882 | 41.6812613 |
| *Canis aureus* | 42.8564101 | 42.2788938 |
| *Canis aureus* | 45.5298794 | 41.9729837 |
| *Canis aureus* | 42.301014 | 42.6030776 |
| *Canis aureus* | 40.1319884 | 43.5179653 |
| *Canis aureus* | 42.6090742 | 41.6407387 |
| *Canis aureus* | 35.9980245 | 47.2116646 |
| *Canis aureus* | 37.2642738 | 48.1055118 |
| *Canis aureus* | 35.5072892 | 46.7855299 |
| *Canis aureus* | 37.091141 | 47.0072521 |
| *Canis aureus* | 37.9914321 | 47.3039646 |
| *Canis aureus* | 37.6314072 | 47.7204569 |
| *Canis aureus* | 36.3752527 | 46.8327549 |
| *Canis aureus* | 35.8663774 | 45.3781218 |
| *Canis aureus* | 35.2746723 | 46.2473635 |
| *Canis aureus* | 39.1904862 | 47.8797544 |
| *Canis aureus* | 47.0915127 | 39.2174433 |
| *Canis aureus* | 45.7061493 | 38.8931912 |
| *Canis aureus* | 44.5032109 | 39.6901158 |
| *Canis aureus* | 47.6855414 | 39.5726891 |
| *Canis aureus* | 44.6771497 | 40.8167702 |
| *Canis aureus* | 45.7556275 | 39.7344426 |
| *Canis aureus* | 46.4662238 | 39.0009689 |
| *Canis aureus* | 43.7380056 | 40.7891071 |
| *Canis aureus* | 46.2680893 | 39.4741715 |
| *Canis aureus* | 44.468006 | 40.3066372 |
| *Canis aureus* | 45.1731884 | 40.2067963 |
| *Canis aureus* | 45.3191779 | 40.7035482 |
| *Canis aureus* | 44.2144994 | 41.1749864 |
| *Canis aureus* | 45.5004486 | 39.3229223 |
| *Canis aureus* | 47.3506078 | 41.2201942 |
| *Canis aureus* | 47.8685872 | 40.3734074 |
| *Canis aureus* | 46.6406734 | 40.0234732 |
| *Canis aureus* | 45.8791937 | 40.4775375 |
| *Canis aureus* | 48.7854206 | 41.4929756 |
| *Canis aureus* | 47.3909196 | 40.0459813 |
| *Canis aureus* | 49.4728655 | 40.6582338 |
| *Canis aureus* | 48.6765953 | 40.0210009 |
| *Canis aureus* | 45.1913972 | 41.337877 |
| *Canis aureus* | 49.1150106 | 41.0209219 |
| *Canis aureus* | 46.4425944 | 41.5429431 |
| *Canis aureus* | 46.2454022 | 40.8885959 |
| *Canis aureus* | 45.2214758 | 43.4810084 |
| *Canis aureus* | 44.5782632 | 44.3878811 |
| *Canis aureus* | 39.0834831 | 46.102694 |
| *Canis aureus* | 39.2533589 | 46.5621568 |
| *Canis aureus* | 44.3511874 | 44.8337396 |
| *Canis aureus* | 37.2339492 | 44.9922164 |
| *Canis aureus* | 44.1089287 | 43.982369 |
| *Canis aureus* | 45.9124815 | 44.2913456 |
| *Canis aureus* | 45.9307479 | 43.3614358 |
| *Canis aureus* | 48.003435 | 41.9209567 |
| *Canis aureus* | 47.2149802 | 42.7330622 |
| *Canis aureus* | 38.3454543 | 46.5676717 |
| *Canis aureus* | 39.5189658 | 47.2496818 |
| *Canis aureus* | 39.1657748 | 44.0783077 |
| *Canis aureus* | 46.7032763 | 44.248796 |
| *Canis aureus* | 45.1425461 | 43.9889549 |
| *Canis aureus* | 39.8708187 | 46.7850726 |
| *Canis aureus* | 45.4874577 | 44.8317341 |
| *Canis aureus* | 46.6410776 | 43.7190433 |
| *Canis aureus* | 39.8397386 | 47.6731783 |
| *Canis aureus* | 37.7210867 | 44.6712891 |
| *Canis aureus* | 38.5287214 | 45.7059739 |
| *Canis aureus* | 45.7797042 | 43.8042215 |
| *Canis aureus* | 38.7884328 | 47.4790996 |
| *Canis aureus* | 46.4286427 | 43.0621996 |
| *Canis aureus* | 22.6177688 | 37.4116828 |
| *Canis aureus* | 23.0280824 | 36.6854407 |
| *Canis aureus* | 21.7416589 | 36.8871679 |
| *Canis aureus* | 22.4401256 | 36.8253993 |
